# Ikaros Proteins in Tumor: Current Perspectives and New Developments

**DOI:** 10.3389/fmolb.2021.788440

**Published:** 2021-12-07

**Authors:** Ruolan Xia, Yuan Cheng, Xuejiao Han, Yuquan Wei, Xiawei Wei

**Affiliations:** Laboratory of Aging Research and Cancer Drug Target, State Key Laboratory of Biotherapy, National Clinical Research Center for Geriatrics, West China Hospital, Sichuan University, Chengdu, China

**Keywords:** Ikaros, Aiolos, hematological malignancies, targeted therapy, immunotherapy

## Abstract

Ikaros is a zinc finger transcription factor (TF) of the Krüppel family member, which significantly regulates normal lymphopoiesis and tumorigenesis. Ikaros can directly initiate or suppress tumor suppressors or oncogenes, consequently regulating the survival and proliferation of cancer cells. Over recent decades, a series of studies have been devoted to exploring and clarifying the relationship between Ikaros and associated tumors. Therapeutic strategies targeting Ikaros have shown promising therapeutic effects in both pre-clinical and clinical trials. Nevertheless, the increasingly prominent problem of drug resistance targeted to Ikaros and its analog is gradually appearing in our field of vision. This article reviews the role of Ikaros in tumorigenesis, the mechanism of drug resistance, the progress of targeting Ikaros in both pre-clinical and clinical trials, and the potential use of associated therapy in cancer therapy.

## 1 Introduction

Ikaros is a zinc finger transcription factor (TF) and a member of the Krüppel family, which is called the IKAROS family zinc finger protein family (IKZF) and consists of other TFs named Ikaros, Helios, Aiolos, Eos, and Pegasus. Ikaros is encoded by the IKZF1 gene (Zhao et al., 2020), exerting an essential effect on regulating normal lymphopoiesis and functions as a tumor suppressor (Winandy et al., 1995; Sigvardsson, 2018). It covers four zinc fingers at the N-terminal for binding to DNA by directly combining with the GGGAA core motif *in vitro* and at the A/GGAAA core motif *in vivo*. At the C-terminal of Ikaros, two additional zinc fingers are required to form homo- and hetero-dimerization between isoforms. The mutations in IKZF are associated with recurrent infections, cytopenia (neutropenia, immune thrombocytopenia, and autoimmune hemolytic anemia), autoimmune diseases, and hematological malignancies (Kuehn et al., 2020).

Current knowledge of the Ikaros family suggests that these TFs are primarily concerned with the development of lymphocytes (Heizmann et al., 2018), covering extensive cellular processes like proliferation, differentiation, cell cycle arrest, and apoptosis (Fan and Lu, 2016). Nevertheless, the absence of Ikaros proteins results in a detrimental production of B lymphocytes, T lymphocytes, NK cells, and dendritic cells (Georgopoulos, 2002; Hariri and Hardin, 2020). Germline mutation in IKZF1 has also been reported to be associated with congenital pancytopenia (Goldman et al., 2012).

### 1.1 Classification and Genome

The Ikaros protein family includes five members named Ikaros, Helios, Aiolos, Eos, and Pegasus. Ikaros, Helios, and Aiolos are principally expressed in some hematopoietic cells and lymphoid cells; notwithstanding, Ikaros is also found in the brain. Eos and Pegasus are widely detected throughout the body, including the brain, liver, skeletal muscle, kidney, and heart (Fan and Lu, 2016) (Table 1). Various IKZF protein TFs emit diverse effects in the maintenance of normal physiological activities or progression of some diseases.

**TABLE 1:** Classification of Ikaros family proteins.

Members	Alias	Distribution	Key function	Related diseases
Ikaros	IKZF1	Hematopoietic system	1). exerts function during specific stages of lymphocyte development	ITP, PIH, SLE, asthma, type 1 diabetes, IBD, sjogren’s syndrome, antiphospholipid syndrome, systemic sclerosis, BCP-ALL, MM, ALL, MCL, CLL, CML, lung cancer, ovarian cancer, HCC, CRC
			2). abnormal expression leads to the occurrence and development of some autoimmune diseases, hematological malignancies, and solid tumors	
Helios	IKZF2	Hematopoietic system	1). strengthens and represents fetal Treg differentiation	RA, SLE, type 1 diabetes, IBD, di George syndrome, HT, PD, AML, BCP-ALL, ALL, hypertension, gastric cancer
			2). abnormal expression is related to autoimmune diseases, hematological malignancies, and solid tumors	
			3). associated with specific infection	
Aiolos	IKZF3	Hematopoietic system	1). of importance for *trans*-differentiation of innate lymphoid	Graves’ disease, SLE, RA, MCL, MM, CLL, ALL, NCC, lung cancer
			2). abnormal expression is related to autoimmune diseases, and solid tumors	
			3). up-regulates cancer stem cell-like properties	
Eos	IKZF4	Non-hematopoietic system	1). leads to gene silencing in Tregs	IBD, EAE, type 1 diabetes, T-CLL
			2). selective deletion leads to systemic autoimmunity	
			3). associated with specific infection	
Pegasus	IKZF5	Non-hematopoietic system	1). related to megakaryopoiesis	thrombocytopenia

Abbreviation: ITP, immune thrombocytopenia; PIH, presumed autoimmune hepatitis; SLE, systemic lupus erythematosus; IBD, inflammatory bowel disease; BCP-ALL, pediatric B-cell precursor acute lymphoblastic leukemia; MM, multiple myeloma; ALL, acute lymphoblastic leukemia; MCL, mantle cell lymphoma; CLL, chronic lymphocytic leukemia; CML, chronic myelogenous leukemia; HCC, hepatocellular carcinoma; CRC, colorectal cancer; RA, rheumatoid arthritis; HT, Hashimoto thyroiditis; PD, Parkinson disease; NCC, nasopharyngeal carcinoma; EAE, experimental autoimmune encephalomyelitis.

#### 1.1.1 Ikaros

**FIGURE 1:**
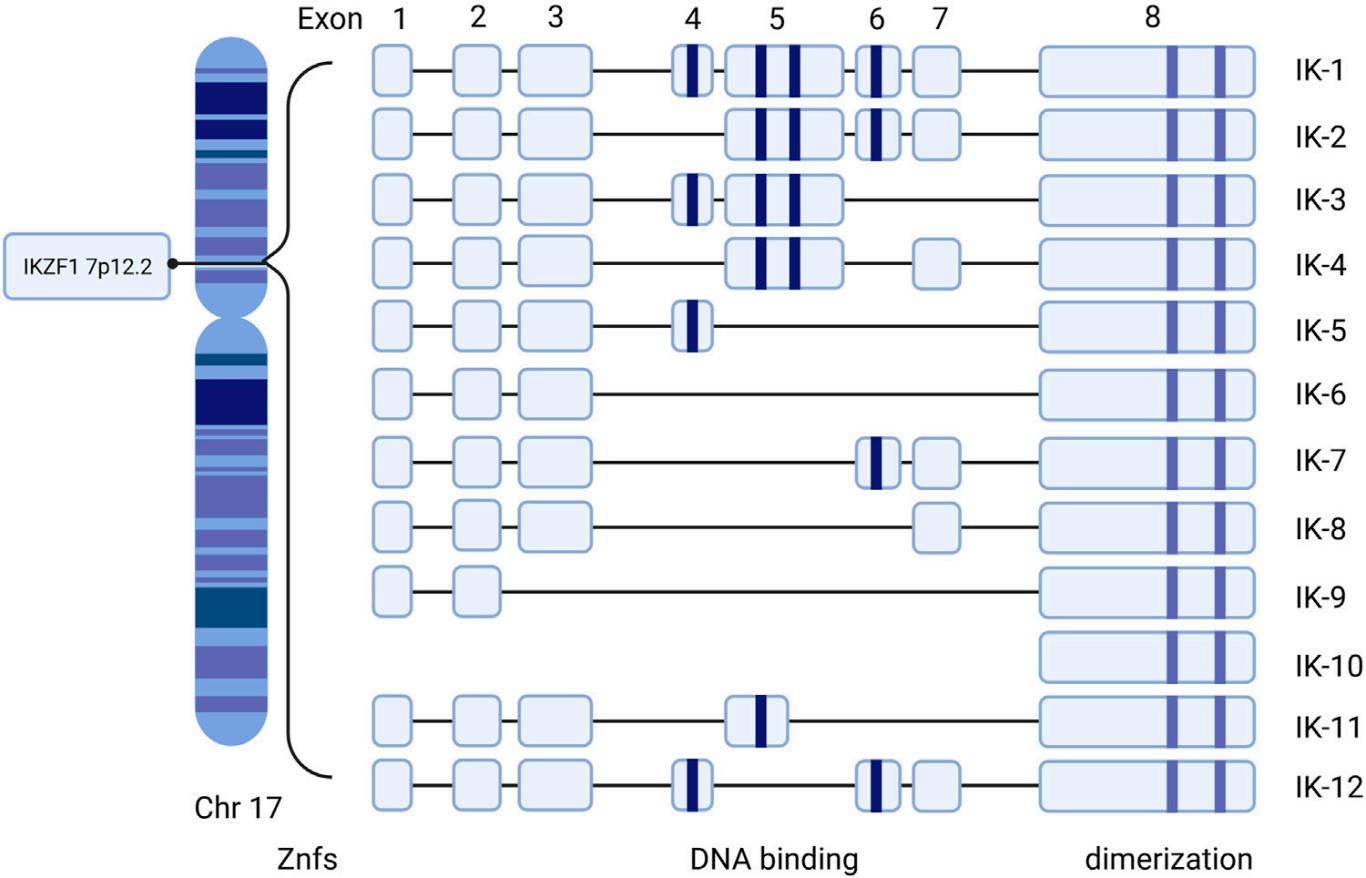
Human Ikaros isoforms produced by IKZF1 transcription. Exons 1–8 refer to the encoding exons. The N-terminal zinc fingers are shown in blue column bars and C-terminal zinc fingers are shown in purple column bars. Znfs: zinc-fingers.

The IKZF1 gene that encodes Ikaros is located on chromosome 7 at 7p12.2 (HGNC, 2021). It consists of 8 exons and codes 519 amino acids. Exon 8 at the C-terminal includes the two domain zinc fingers required to form homo- and hetero-dimerization and four N-terminal DNA-binding zinc fingers for binding to the core motif at DNA (Ruiz et al., 2004; Zhang et al., 2020; Payne, 2011). Different combinations of zinc finger modules influence the capacity of DNA-binding and functional properties (Molnár and Georgopoulos, 1994; Vairy and Tran, 2020). At least 12 isoforms, including Ik1–12, are generated through alternative splicing Ikaros genes encoding a zinc finger protein with eight exons (Molnár and Georgopoulos, 1994; Yamamoto et al., 2005; Hahm et al., 1994) (Figure 1). Among all the isoforms, the largest isoforms are Ik1 or Ik-H, which have four zinc finger domains at the N-terminal and two at the end of the C-terminal (Ronni et al., 2007). In addition, dimerization among Ikaros isoforms either enhances or suppresses its affinity of DNA binding, thus affecting the general Ikaros transcriptional activity through specific mechanisms such as chromatin-remodeling complexes and epigenetic modification (Georgopoulos et al., 1997; McCarty et al., 2003).

Isoforms with at least three DNA binding domains can combine with Ikaros conservative DNA binding sites, while isoforms with less than three DNA binding domains are not able to bind these motifs and remain in the cytoplasm (Hahm et al., 1994). Hence, the functional characteristics and subcellular localization of Ikaros isotypes may be different. Besides, the expression of Ikaros is correlated to the morphology of the nucleus. When cytogenetic outcomes are not available, the morphology of the cuplike nucleus can help to indicate the presence of IKZF1 deletion at a high predictive value (Li et al., 2019). IK6, due to the deficiency of exons 4–7, lacks four N-terminal zinc fingers but retains the C-terminal dimerization domain, which is most closely related to oncogenicity (Vairy and Tran, 2020). The presence of certain isoforms may be associated with the occurrence and prognosis of corresponding clinical diseases. Ik6 and Ik10 are highly related to a poor clinical outcome in B-lineage acute lymphoblastic leukemia (ALL) in children (Moreira et al., 2019). There is a correlation between Ik6 expression and t (4; 11) translocation in patients with leukemia (Ruiz et al., 2004; Zhang et al., 2020).

#### 1.1.2 Helios

Helios, also called IKZF2, is of immediate clinical significance due to the fact that it plays a similar function to Ikaros in regulating the functional property of immune cells and the progression of hematological malignancies. Helios is a marker of stable and inhibitory T regulated cells (Tregs) (Thornton and Shevach, 2019), acting as an essential part to preferentially strengthen the differentiation of fetal Tregs and fine-adjusting of fetal Tregs (Ng et al., 2019). Helios-deficient Tregs show an unstable phenotype in the process of inflammation, characterized by decreased expression of FoxP3 and increased expression of effector cytokines following weakened activation of the STAT5 pathway (Kim et al., 2015).

In addition, the high expression of IKZF2 and low level of interleukin-7 receptor (IL7R) are identifiable transcriptional spectra demonstrated in CD16 ^+^ CD8 T cells, which are related to chronic untreated HIV-1 infection (Naluyima et al., 2019). Furthermore, Helios was found to be expressed in acute myeloid leukemia (AML) cells, and its depletion in AML cells resulted in decreased colony formation and slowed down oncogenesis (Park et al., 2019). The elevating Helios levels are related to the capacity of Tregs in malignant pleural effusion due to the downregulation of miR-4772-3p (Yu et al., 2019).

#### 1.1.3 Aiolos

Aiolos, encoded by the IKZF3 gene, is located in 17q11.2∼21 and made up of eight exons and seven introns (Hosokawa et al., 1999). The *trans*-differentiation from innate lymphoid cell 3 (ILC3) to ILC1/NK cells is linked with high expression of Aiolos and can be reduced through lenalidomide (Len) targeting Aiolos and Ikaros (Mazzurana et al., 2019). In addition, it is of great importance in the occurrence, metastasis, and prognosis of certain hematological malignancies and solid tumors (Duhamel et al., 2008; Li et al., 2014). Aiolos has been found to be expressed in lung cancer cells ectopically, which instigates cancer metastasis through reducing expression of many adhesion-related genes, spoiling cell-cell or cell-matrix interactions, and promoting anchorage independence (Wang et al., 2019; Li et al., 2014). Moreover, solid tumors are able to mimic the cellular behavior related to lymphocyte transport during immune surveillance through the “identity theft” of hematopoiesis led by the expression of Aiolos (Li et al., 2014). In addition to increasing invasive and migratory ability in lung cancer cells through upregulation of the phosphatidylinositol-3 kinase (PI3K)/AKT/Twist axis induced by Aiolos overexpression, the overexpression of Aiolos also upregulates cancer stem cell-like properties through the same pathway (Hung et al., 2019).

#### 1.1.4 Eos

Eos, a novel transcriptional regulator encoded by the IKZF4 gene, is required for the suppressive function of Treg cells *in vivo* (Gokhale et al., 2019). Eos directly interacts with Foxp3, leading to chromatin modifications, which results in gene silencing in Tregs (Pan et al., 2009). While Tregs of mice with an overall absence of Eos were demonstrated to have completely normal suppressive function both *in vivo* and *in vitro* (Rieder et al., 2015; Gokhale et al., 2019). In peripheral blood mononuclear cells of individuals with HTLV-1 infection, the aberrant expression of Eos may correlate to the pathological progression of HTLV-1-related adult T-cell leukemia/lymphoma and myelopathy/tropical spastic paraparesis (Naito et al., 2019).

#### 1.1.5 Pegasus

Pegasus is a novel transcriptional regulator encoded by IKZF5, with an unclear physiological function of normal hematopoiesis. It was reported that Pegasus was related to megakaryopoiesis and dominant thrombocytopenia in humans (Lentaigne et al., 2019).

### 1.2 Ikaros Family Proteins and Related Diseases

Ikaros proteins are identified to serve as suppressors in diverse types of lymphoma or leukemia (Heizmann et al., 2018; Chan, 2019), whereas they are overexpressed in other malignancies to maintain cancer cell proliferation and survival, such as in malignant plasma cells, monoclonal gammopathy of undetermined significance, and multiple myeloma (MM) (Cippitelli et al., 2021). Detailed functional analyses showed that Ikaros could excite or suppress oncogenes or tumor suppressors genes; consequently, Ikaros-mediated transcriptional expression of target genes regulates survival and proliferation of cancer cells (Gowda et al., 2017a). Specifically, Ikaros was reported to control human skin fibroblast cell migration negatively by GSK3β-Ikaros-ANXA4 signaling (Wang et al., 2020a).

It is assumed that Ikaros family deficiency may lead to a variety of immune-associated diseases, including immune thrombocytopenia (Sriaroon et al., 2019), presumed autoimmune hepatitis (Groth et al., 2020), systemic lupus erythematosus (SLE) (Cunninghame Graham et al., 2011; Jeng et al., 2019; Chen et al., 2020), rheumatoid arthritis (Yang et al., 2019), asthma (Igartua et al., 2015), type 1 diabetes (Davidson and Diamond, 2001; Swafford et al., 2011; Lempainen et al., 2013; Khamechian et al., 2018), Graves’ disease (Li et al., 2018a), Hashimoto thyroiditis (Hu et al., 2019), inflammatory bowel disease (Crohn disease) (Barrett et al., 2008; Eskandarian et al., 2019; Sznurkowska et al., 2020), di George syndrome (Klocperk et al., 2014), antiphospholipid syndrome (Dieudonné et al., 2019), Parkinson disease (Daneshvar Kakhaki et al., 2020), Sjogren’s syndrome, and systemic sclerosis (Gorlova et al., 2011).

It also shows that Ikaros can manage myeloid cell proliferation, and somatic Ikaros mutations are related to myeloproliferative disorders (Theocharides et al., 2015). In addition, the occurrence and maintenance of numerous human cancers, such as pediatric B-cell precursor acute lymphoblastic leukemia (BCP-ALL) (Churchman et al., 2018; Stanulla et al., 2018; Tayel et al., 2019), lung cancer (Li et al., 2014; Zhao et al., 2020), breast cancer (Edgren et al., 2011), nasopharyngeal carcinoma (Verhoeven et al., 2019), ovarian (He et al., 2012), liver (Liu et al., 2017), and colorectal cancer (Javierre et al., 2011), are also correlated with the abnormal expression of Ikaros family proteins. Recently, for some solid tumors, it was shown that a higher level of Ikaros is correlated with poor differentiation and advanced stage of ovarian cancer (He et al., 2012), while it functions as an anticancer character in hepatocellular carcinoma through inhibiting CD133 and ANXA4 expression (Liu et al., 2017). Besides, the hypermethylation of Ikaros levels could be considered as a sign of the progression of colorectal cancer (CRC) and inform adequacy of surgical resection about CRC (Javierre et al., 2011; Symonds et al., 2018; Symonds et al., 2020).

For patients with MM, the Ikaros family proteins served as predictors of prognosis for MM patients treated by Len (Kriegsmann et al., 2019; Tachita et al., 2020). However, a correlation between the expression of IKZF1 or IKZF3 and patients’ reaction to Len from immunohistochemical analysis remains obscure (Dimopoulos et al., 2019). It is considered that the effect of low IKZF1 or IKZF3 levels on the adverse outcome of Len therapy results in shorter progression-free survival and overall survival (Zhu et al., 2014; Pourabdollah et al., 2016; Dimopoulos et al., 2019).

In ALL, approximately 50% of adult patients possess IKZF1 genetic mutations, including beyond 80% of patients with BCR-ABL1-positive (Ph+) ALL. A total of 15% of IKZF1 genetic alteration can be found in childhood B-cell ALL, covering about 70% of Ph + ALL patients (Mullighan et al., 2007; Mullighan et al., 2008). Patients with ALL have Ikaros mutations with characteristic resistance to treatment (Marke et al., 2016), high relapse rate (Kuiper et al., 2010; Berry et al., 2020), and poor prognosis (Mullighan et al., 2009; Aref et al., 2020). Genetic and functional abnormalities of IKZF1, including deletion of a single Ikaros, were regarded as new prognostic indicators for high-risk leukemia in clinical trials (NCT00993538; NCT03709719, NCT01431664) (Mi et al., 2012; Tang et al., 2019; Granados-Zamora et al., 2020).

In chronic myelogenous leukemia (CML), the deletions in IKZF1 and codeletion of other genes are identified in a chronic phase CML diagnostic sample (Klumb et al., 2019). Moreover, the deficiency or reduction of Ikaros is deemed as a common step and potential diagnostic precursor of progressive myeloid disease in patients with CML (Beer et al., 2015).

Immunomodulatory drugs (IMiDs), consist of thalidomide, Len, pomalidomide, and an analog, target a ubiquitous protein called CRBN to induce the degradation of Ikaros. The efficacy and safety of those drugs have been verified in a wide range of clinical trials, and it is increasingly clear that the efficacy of IMiDs in the treatment of MM (Gao et al., 2020), myelodysplastic syndrome (MDS) with deletion of chromosome 5q (Fenaux et al., 2011), mantle cell lymphoma (MCL) (Ruan et al., 2018) and chronic lymphocytic leukemia (CLL) (Vitale et al., 2016; Zhou et al., 2020) is promising. Despite the development of Ikaros-targeted therapy, the incidence of drug resistance is increasing. Herein, we summarized the molecular characteristic of Ikaros, the mechanism of the Ikaros-associated pathway, and recent anti-Ikaros drug development based on clinical trials in our review.

## 2 Ikaros Family Signaling

Ikaros seems to act both as a transcriptional repressor and as an activator by binding to assorted nuclear factors referred to as epigenetic regulation and chromatin remodeling. If recruiting histone remodeling complexes such as nucleosome remodeling and deacetylase complex (NuRD) *via* direct binding to Mi-2, it will mediate tumor inhibition. If integrating into the ATP-dependent chromatin remodeling complexes SW1/SNF, it will cause gene activation (Dhanyamraju et al., 2020; Payne et al., 2020). Ikaros also directly engages with and recruits distinct histone deacetylase complexes (HDAC1 and HDAC2) to specific promoters of its target genes to modulate gene expression and to exert tumor-suppressive effects (Koipally et al., 1999; Song et al., 2016). Ikaros participates in a NuRD complex with acetyltransferases, methyltransferases, deacetylases, and the chromatin remodeling complex (Oliveira et al., 2019). In addition to the NuRD complex, the positive-transcription elongation factor b and the protein phosphatase 1α (PP1) are required to assist transcription extension of Ikaros target genes and regular differentiation of hematopoietic progenitor cells (Bottardi et al., 2014). In addition, Ikaros manipulates cellular proliferation by means of suppressing the PI3K pathway and genetic expression that promote cell cycle progression (Song et al., 2015).

### 2.1 Signaling Pathways About Ikaros Family

**FIGURE 2:**
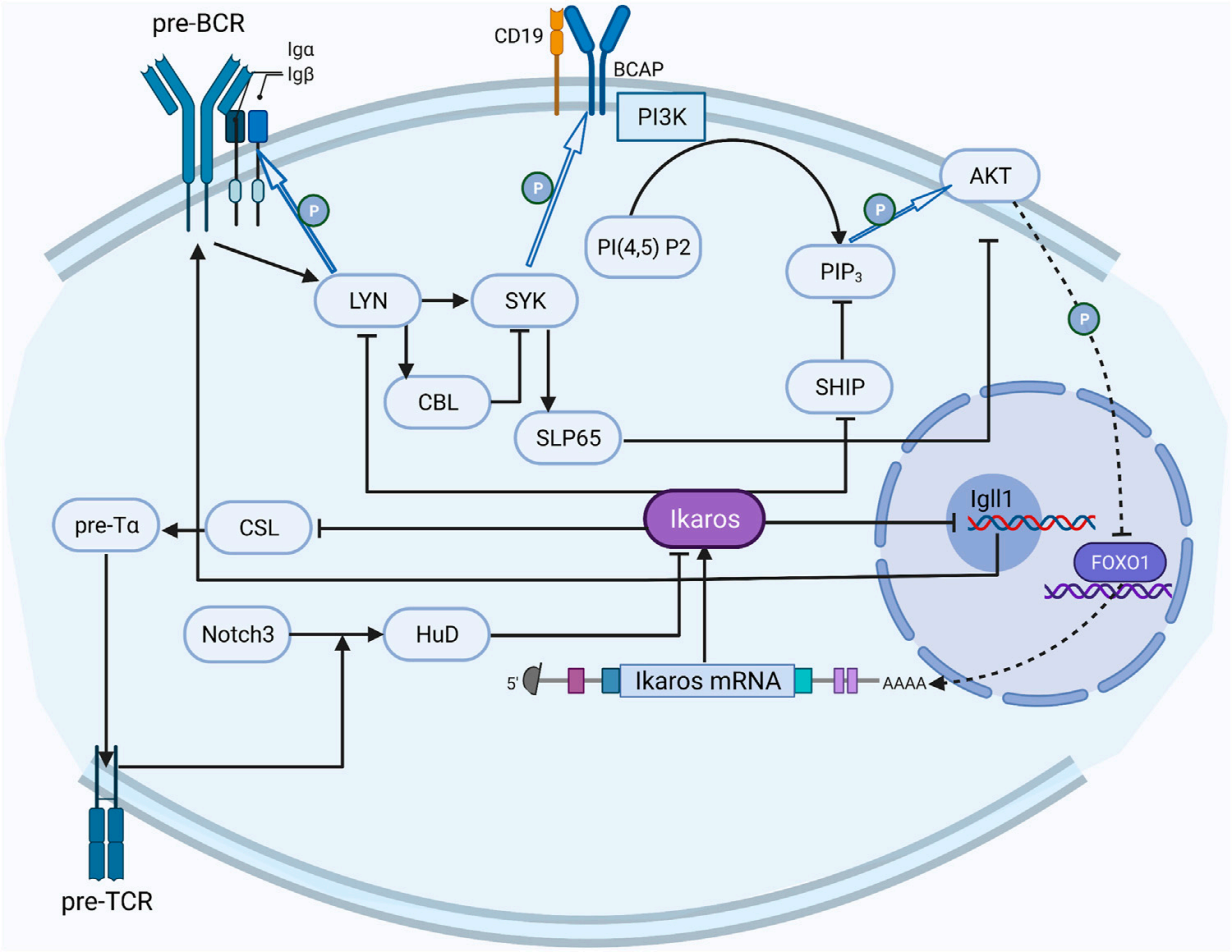
The regulation network of Ikaros. The activated preBCR pathway decreases the activity of Ikaros, Ikaros counteracts this inhibition by repressing LYN and SHIP in the pathway; Notch3 upregulates HuD to convert Ikaros to the dominant negative isoform, and Ikaros counteracts the Notch effect on CSL activation through competing with the CSL DNA binding site in the promoter region.

Studies on signaling pathways about Ikaros have attracted some attention (Figure 2). The most fully studied Ikaros-related pathway is the preBCR (B-cell receptor) signal pathway, which has formed a distinct picture of the regulatory network (Alkhatib et al., 2012). Reciprocally, the activated preBCR pathway eventually decreased the activity of Ikaros; Ikaros can counteract this effect by suppressing two sites in the pathway (Yasuda et al., 2000; Nera et al., 2006; Nakayama et al., 2009).

The Notch pathway has also been relatively well studied. Notch3 converts the alternative splicing mode of Ikaros to a dominant-negative isoform by upregulating the expression of RNA binding protein HuD (Bellavia et al., 2007). On the contrary, Ikaros counteracts the Notch effect on CSL activation through competing with the CSL DNA binding site in the promoter region and inhibits the expression of the downstream genes, including the components of preTCR (Dumortier et al., 2006).

It was regarded that ERK1/2-mediated ETS1 phosphorylation decreased the ability of ETS1 to increase Ikaros expression (Joshi et al., 2014). And the activated integrin signaling pathway was reported to be restrained by Ikaros as well. Ikaros is affected by several interferon regulatory factors (IRFs), among which IRF4 and IRF8 were considered to induce the expression of Ikaros and its homologous gene Aiolos (Ma et al., 2008); however, another study showed that IRF8 rather than IRF4 triggers the IKZF1 promoter, and IRF5 could inhibit this activation (Fang et al., 2012).

### 2.2 Activation of Ikaros

Ikaros activities are thought to be regulated by post-translational phosphorylation, small ubiquitin-related modifier (SUMOylation), and ubiquitination (Sridharan and Smale, 2007). Besides, the hypomethylated pattern of CpG island in the IKZF1 promoter region may be the basis of abnormal Ikaros expression patterns associated with malignant tumors (Chen et al., 2019; Rahmani et al., 2019). SUMOylation interferes in the interaction of Ikaros with transcriptional co-repressors SIN3A, SIN3B, Mi-2β, and CtBP and weakens the inhibitory activity of Ikaros (Gómez-del Arco et al., 2005). And the participations of Ikaros in HDAC-dependent and HDAC-independent inhibition are disrupted by Ikaros SUMOylation, but the nuclear localization to pericentromeric heterochromatin is not affected (Gómez-del Arco et al., 2005). Ikaros SUMOylation was discovered to exist in B-ALL cells, whereas it did not show up in normal peripheral blood leukocytes, indicating its potential work in leukemia (Chen et al., 2019). The process of deSUMOylation was actively modulated by SUMO-specific protease Senp1, Axam, and yeast Ulp1 (Ihara et al., 2007). Certainly, Ikaros is ubiquitinated by E3 ligase CRBN7 and degraded by proteasome under the induction of IMiDs, while inhibition of Ikaros ubiquitination is correlated to interact with some TFs like runt-related transcription factor families (RUNXs) (Zhou et al., 2019; Liu et al., 2021). Otherwise, the absence of the E120 enhancer led to an evidential decrease in Ikzf1 mRNA. Nevertheless, the epigenetic pattern and 3D topology of this locus are only slightly impacted, emphasizing the complicacy of the regulatory pattern of the Ikzf1 locus (Alomairi et al., 2020).

The effect of carcinogenic casein kinase II (CK2) on the phosphorylation of Ikaros has been widely studied. CK2 is a multipotent serine/threonine kinase, which is overexpressed in various cancers, including leukemia (Cunninghame Graham et al., 2011; Jeng et al., 2019; Chen et al., 2020). Studies have shown that CK2 directly phosphorylates multiple amino acids in the whole Ikaros protein, and hyperphosphorylated Ikaros facilitates self-degradation through the ubiquitin/proteasome pathway (Dovat et al., 2011). The application of phosphomimetic esters and phosphoresistant Ikaros mutants found that the phosphorylation of CK2 phosphate sites seriously decreases the ability of Ikaros to bind to DNA and alters the localization to pericentromeric heterochromatin, resulting in the dysfunction of Ikaros proteins (Gurel et al., 2008). Pharmacological inhibition of CK2 can restore the DNA binding ability and tumor inhibitory activity of Ikaros and cause leukemia cytotoxicity in the high-risk model of xenotransplantation in patients with ALL, highlighting the fact that CK2 inhibitors can be used as potential therapeutic strategies for high-risk pediatric leukemia (Song et al., 2015; Gowda et al., 2017a). Ikaros phosphorylation by CK2 is cell periodicity, indicating that CK2 effects the regulation of Ikaros function during G1/S transition and S phase in human leukemia (Arco et al., 2004; Li et al., 2012). Besides, CK2-mediated phosphorylation of Ikaros was vital to regulate the transcriptionally of the terminal deoxy transferase gene during differentiation of thymocytes (Wang et al., 2014). SYK is able to phosphorylate Ikaros at dissimilar sites, affecting Ikaros’ nuclear localization (Uckun et al., 2012).

CK2-mediated phosphorylation is reversed by PP1 to dephosphorylate Ikaros (Popescu et al., 2009; Song et al., 2011). The mutation of the PP1 interaction site of Ikaros or the pharmacological inhibition of PP1 lead to the hyperphosphorylation of Ikaros, which seriously reduces the DNA binding ability of Ikaros, loses the pericentromeric localization of Ikaros, and increases degradation of Ikaros through the ubiquitous protein pathway (Popescu et al., 2009).

### 2.3 Molecular Mechanisms of Ikaros in Immune Cells

**FIGURE 3:**
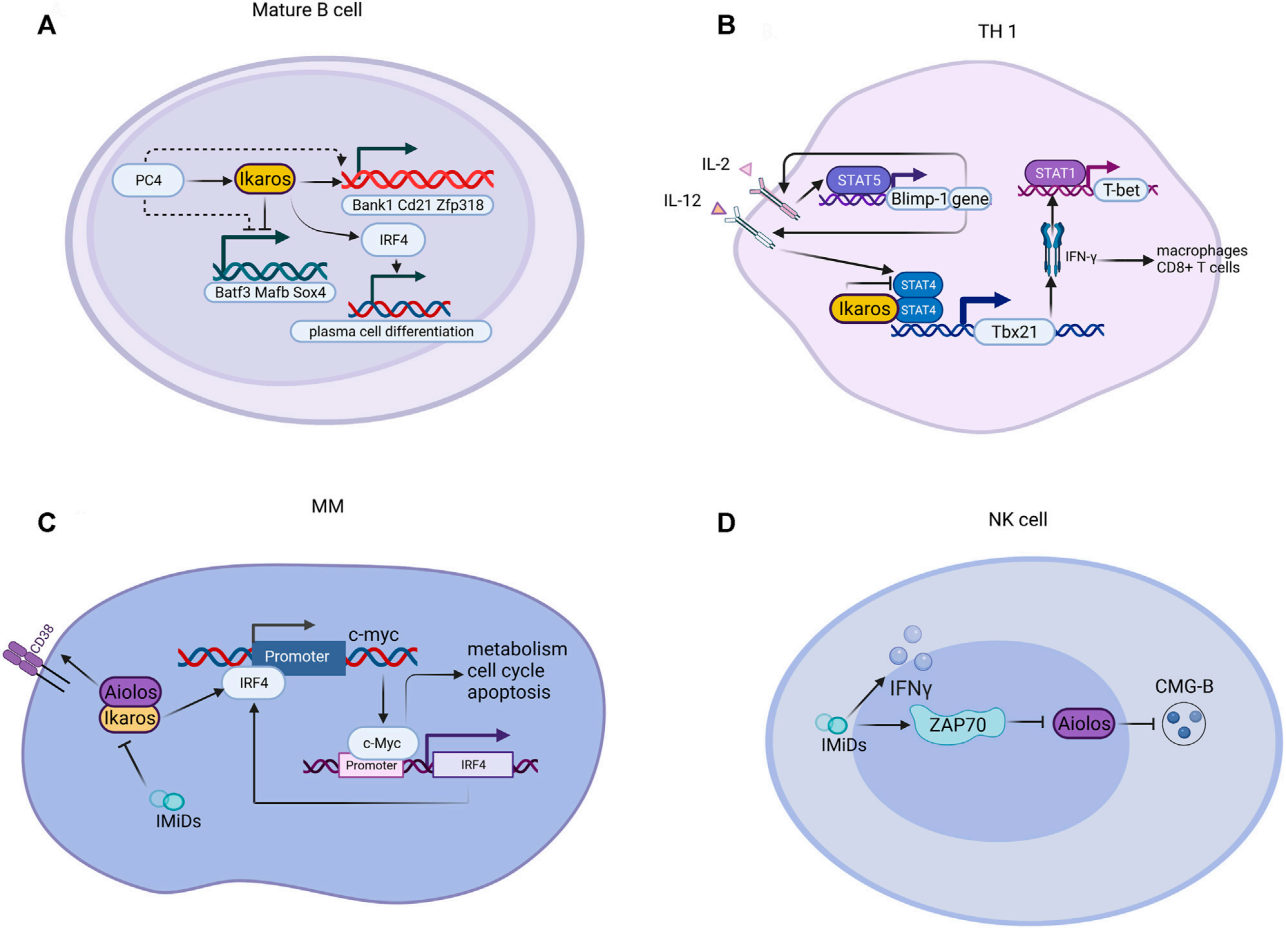
Illustration of genes regulated by Ikaros in tumors. Ikaros is a tumor suppressor gene, controlling the transcription of ma genes related to leukemogenesis.

Ikaros has different effects on the growth, reproduction, and differentiation of many kinds of innate or adaptive lymphocytes *in vivo* (Figure 3). It has been previously reported that Ikaros is vital for the conversion between the large and small pre-B stages (Schwickert et al., 2014). Ikaros also sustains B-cell proliferation and differentiation through initiating kinase-signaling cascades and collaborating with chromatin protein 4 (Ochiai et al., 2020). In addition, the plasma cell mal-differentiation of sub1 deficient B cells can be saved by Ikaros and IRF4 (Ochiai et al., 2020). For innate lymphoid cells, Ikaros, the significant regulator of ILC3 existence and function, represses the transcriptional activity of aryl hydrocarbon receptors in a zinc finger-dependent manner, inhibits ILC3 in a cellular manner, and controls intestinal immune response in steady-state and disease (Li et al., 2016). Ikaros and Aiolos play a critical role in regulating the *trans*-differentiation of ILC3-ILC1/NK cells (Bald et al., 2019).

IKZF1 was demonstrated to regulate embryonic T lymphopoiesis of zebrafish via chemokine receptor 9 and IRF4 (Huang et al., 2019). Members of the IKZF family are also involved in the differentiation and property of single T helper cell subsets, covering TH1, TH2, TH17, T follicular, and Tregs (Powell et al., 2019). The function of Ikaros protein in T cell differentiation has been revealed, whereas contradictory results were obtained in certain mouse models. Research utilizing T cells possessing germline Ikaros gene mutations demonstrated that Ikaros promotes Th17 and Treg cell differentiation and suppresses the polarization of Th1 (Cippitelli et al., 2021). Nevertheless, in a mouse model in which Ikaros conditioned knockout in mature T cells, the deficiency of Ikaros is related to the acquirement of Th1, Th2, Th17 but not Treg cells (Lyon de Ana et al., 2019).

Ikaros supports signal-induced downregulation of recombination-activating gene 1 (RAG1) and RAG2 gene expression in CD4 ^+^ CD8^+^ positive thymocytes through a non-redundant manner (Naik et al., 2019). Ikaros plays a role in preventing autoimmunity by administering BCR unresponsiveness and repressing TLR signaling transduction (Schwickert et al., 2019). Although lymphocytes need Ikaros to differentiate, the role of the Ikaros protein family in the myeloid cell is not clear (Park et al., 2015). The mouse model has shown that Ikaros participates in the regulation of differentiation of neutrophils by silencing permissible or specific pathways in the ordinary precursors of macrophage-monocyte evolution (Dumortier et al., 2003). Ikaros modulates early phase differentiation of neutrophils but is optional for mature neutrophils (Dumortier et al., 2003).

### 2.4 Tumorigenesis and Antitumor Effect of Ikaros

**FIGURE 4:**
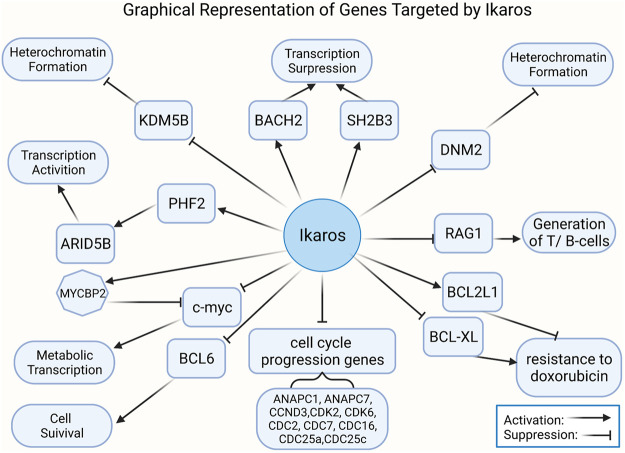
Mechanism of Ikaros family proteins in various cells. **(A)** For mature B cells, PC4 increases and cooperatively assists Ikaros proteins to regulate gene expression; the PC4-IKAROS-IRF4 axis activates genes (e.g., Bank1, Cd21, and Zfp318), represses non-B cell genes (e.g., Batf3, Mafb, and Sox4), and promotes plasma cell differentiation. **(B)** Ikaros suppresses the polarization of Th1 through suppressing transcription mediated by STATA4 and downstream factors. **(C)** IMiDs interfere with disease-promoting activities of c-Myc and IRF4 *via* Aiolos and Ikaros in MM. **(D)** IMiDs bind to ZAP70, downregulate Aiolos, and enhance CMG-B. CMG-B, cytotoxic molecular granzyme B.

Ikaros appears to function as a transcriptional inhibitor and activator by binding to different targets, such as some nuclear factors related to epigenetic regulation (Figure 4). Ikaros suppresses target gene transcription by directly binding or recruiting HDAC1 (Ikaros-HDAC1 complex) to induce the formation of inhibitory chromatin: the former way results in the increase of H3K9me3 and the reduction of H3K9ac, while the latter complex can promote the affinity for DNA binding to the promoter of lysine [K]-specific demethylase 5B (KDM5B) by CK2 inhibitors and raise formation of H3K27me3 and reduce H3K9ac (Song et al., 2016; Wang et al., 2016). The CK2-Ikaros axis also exerts beneficial control over Ikaros target gene expression, increasing PHD finger protein 2 (PHF2) expression, which forms a complex with AT-rich interactive domain-containing protein 5B (ARID5B) to activate the target genes’ transcription (Ge et al., 2018a; Ge et al., 2018b). The phosphorylation by CK2 overexpressed in B-ALL depresses Ikaros combining and recruiting HDAC1 to the promoter of BCL2L1, which causes repression of BCL2L1 and increases expression of BCL-XL (Schott et al., 2020). Recent studies have indicated that Ikaros has a negative effect on the development of T cell leukemia *via* globally regulating the enhancer or super-enhancer landscape and pioneering activity (Ding et al., 2019). Ikaros has a critical role in regulating *de novo* enhancer formation, super-enhancers formation, depletion of enhancers, and stimulation of poised enhancers, demonstrating that Ikaros direct modulates the expression of numerous genes than hypothesized formerly (Gowda et al., 2020). Furthermore, miR-26b expression is induced by the differential expression of Ikaros isoforms and transcriptional regulators of miR-26b modulated by PTEN (Yuan et al., 2017). The negative prognosis of IKZF1 deletion in BCP-ALL may be strengthened by the activation of Janus kinase signal transducer and activator of transcription (JAK/STAT) signaling and recede by ERG deletion (Stanulla et al., 2018). The mutual control of Notch1 and Ikaros in DN2 subsets of the thymus in tumor-bearing mice promotes the early stagnation of T cell development at the DN2a stage and its transfer to dendritic cells lineage (Guha et al., 2020). In terms of metabolism, the expression of functional paired box 5(PAX5) and Ikaros induces the powerful upregulation of glucose-6-phosphate dehydrogenase (G6PD). Interestingly, in clinical trials of patients with lymphoma, patients with high expression of G6PD were linked to poor overall prognosis (Xiao et al., 2018).

These variants of IKZF1 were distributed across the whole gene, particularly located to the C-terminal zinc finger dimerization domain, whereas in immunodeficient patients, germline IKZF1 variants are restricted to the N-terminal zinc fingers (Churchman et al., 2018). IKZF1 mutations and deletions have been suggested to contribute to the occurrence and poor prognosis of ALL and AML (Mullighan et al., 2008; Mullighan et al., 2009; de Rooij et al., 2015; Zhang et al., 2020). Deletion of IKZF1 was also demonstrated as an acquired convert at the transformation phase from CML to lymphoid blast crisis or ALL (Li et al., 2018b). The IKZF1 deletions lead to haploinsufficiency, dominant-negative Ikaros forms, or complete loss of expression of Ikaros. The principle of this dominant-negative effect may be that the high level of mutant unbinding DNA isomers with less than three N-terminal sequences occurs through the formation of heterodimers with wild-type isomers, resulting in alteration in DNA binding properties and them becoming repressors (Vairy and Tran, 2020). Mutations at the C-terminal zinc fingers could also cause a deep effect on the transcription of target genes (Vairy and Tran, 2020). Even after a comprehensive analysis of DNA binding sites of Ikaros in murine hematopoietic cells, the molecular mechanism of tumor suppressor effects in leukemia regulated by Ikaros-mediated transcription is still not clear (Song et al., 2015).

Overexpression of Ikaros leads to inhibition of transcription of genes supporting the PI3K pathway and generates transcription of genes, like INPP5D, repressing the PI3K pathway (Song et al., 2015). And AKT phosphorylation decreasing by overexpression of Ikaros in leukemia cells is identical to the effect of treatment with imatinib (Gowda et al., 2017a). Nevertheless, the JAK/STAT pathways and PI3K/AKT pathways were proved to activate with Ik6 expression in ALL (Song et al., 2015; Qu et al., 2019). Ikaros overexpression also directly suppresses the promoter activity of these clinically significant cell cycle progression genes, including ANAPC1, ANAPC7, CCND3, CDK2, CDK6, CDC2, CDC7, CDC16, CDC25a, CDC25c, and CCNE2 (Dhanyamraju et al., 2020). In addition, KDM5B expression is downregulated by Ikaros through recruiting HDAC1 to the KDM5B gene promoter, which causes an inhibitory chromatin condition and transcriptional inhibition consequently (Dhanyamraju et al., 2020). Ikaros upregulates the PHF2 expression through chromatin remodeling, as demonstrated by the appearance of increased H3K4me3 in the promoter of the PHF2 gene (Ge et al., 2016a; Ge et al., 2018a; Maciel et al., 2019). The expression of ARID5B is positively adjusted by Ikaros, and the loss of a single copy of IKZF1 is associated with low ARID5B expression (Ge et al., 2018b). Gain- and loss-of-function experiments of Ikaros in ALL shows that Ikaros inhibits c-myc gene transcription but controls transcription of MYC binding protein 2 positively, which suppresses the activity of myc (Ge et al., 2015). STAT5 regulates the super-enhancer of the myc gene by means of competition for binding to target sites or regulating histone acetylation in an opposing way to Ikaros (Katerndahl et al., 2017). It was revealed that the genes related to the BCR signal pathway and the IKZF family are presumed to be upstream of the MYC/IRF4 axis (Tsukamoto et al., 2020). In patients with trisomy 12 CLL, IRF4 by means of inducing Ikaros mediates the overexpression of CD49d, which was considered to identify those patients treated with ibrutinib with the characteristics of inferior nodal responses and shorter clinical outcomes (Fiorcari et al., 2019).

Moreover, Ikaros was also verified to negatively regulate IL7R expression and promote SH2B adaptor protein 3 (SH2B3) transcription (Ge et al., 2016b). Ikaros represses B-cell lymphoma 6 (BCL6) transcription and initiates basic leucine zipper transcription factor 2 (BACH2) gene transcription, suggested by BCL6 and BACH2 functional experiments (Ge et al., 2017). Some data identified that Ikaros inhibits dynamin 2 in leukemia by directly combining with the promoter and inducing heterochromatin formation (Ge et al., 2016). PAX5, RUNX1, and IKZF1 were found to overlap abundant target genes, and a modality with dominant-negative effective of IKZF1 or ETV6-RUNX1 fusion protein cooperates with the loss of heterozygosity of PAX5 to regulate gene expression (Okuyama et al., 2019). Furthermore, RAG1 is considered as an immediate target of Ikaros (Li et al., 2016; Dimopoulos et al., 2019). IKZF1 was defined as a crucial *trans*-activator of the SLAMF-7 gene, which is mainly expressed in MM cells and deemed as an ideal target for immunotherapy (Kikuchi et al., 2020).

In contrast to the role of IKZF2 as a tumor suppressor gene in hypodiploid B-ALL, it is essential for AML (Park et al., 2019). The deficiency of IKZF2 in AML cells is related to the reduction of colony formation, augment of differentiation and apoptosis, defective leukemic stem cells function, and tardive leukemogenesis (Park et al., 2015; Park et al., 2019). One of the main mechanisms may be that IKZF2 determines a self-renewal gene expression procedure called HOXA9 and represses a C/EBP-driven differentiation program. In addition, this forced reduction of Ikzf2 is accompanied by decreasing HOXA9 and BCL2, but not Mll, Myc, or Meis1.

## 3 Targeted Therapy for Ikaros

IMiDs, covering thalidomide, Len, and pomalidomide, are clinically approved medicine for the therapy of MM and other malignancies (Table 2). Among them, the relatively well-studied diseases are MM and MDS. These drugs lead to selective ubiquitination and proteasomal degradation of Ikaros and Aiolos through recruiting TFs to the CRL4^CRBN^ E3 ubiquitin ligase, representing an original mechanism of therapy through altering the substrate specificity (Lu et al., 2014; Sievers et al., 2018; Zeidner et al., 2020). Different IMiDs target the degradation of distinct sets of TFs. Furthermore, IMiDs have the ability of direct inhibition of tumor cell growth and strong immunostimulatory characteristics, consequently having multiple implications on the presence of different cellular components in the tumor microenvironment (Cippitelli et al., 2021).

**TABLE 2:** Classification and characteristic of IMiDs.

Description	Mechanism	Indications	Adverse-effect
Thalidomide	1). Anti-angiogenic properties	MM, PCDs, erythema; sarcoidosis, CLE, Behçet’s disease; GVHD; RA, AS, Still’s disease, systemic sclerosis, Sjögren’s syndrome, CD, Kaposi’s sarcoma, CHF, Waldenström’s macroglobulinemia, myelodysplasia, prostate cancer, Renal-cell carcinoma, Glioma, CRC, Melanoma	neuropathy, constipation, sedation, DVT, AIHA, vasculitis
	2). Anti-proliferative effects		
	a). Enhances degradation of Ikaros and Aiolos		
	b). Induces cell cycle arrest		
Lenalidomide	1). Anti-angiogenic properties	MM, FL, MCL, DLBCL, primary CNS/intraocular lymphoma, myelofibrosis, MDS	myelosuppression, skin rash, DVT, interstitial pneumonitis, AIHA, ITP, Evans syndrome, thrombocytopenia, autoimmune thyroiditis, optic neuritis, polymyositis
	2). Anti-proliferative effects		
	a). Enhances degradation of Ikaros and Aiolos		
	b). Induces cell cycle arrest		
	c). Cytoskeletal reorganization		
	d). Inhibition of tumor oncogenes, induction of tumor suppressor genes		
	3). Immunomodulatory		
	a). Increases number of NK cells and NK cell ADCC		
	b). Restores formation of immune synapse		
	c). Suppresses Treg multiplication		
	d). Increases production of IFNγ, IL2 and Th1 cytokine		
	e). Co-stimulation of tyrosine phosphorylation		
	f). Activation of PI3 kinase signaling pathway		
Pomalidomide	1). Anti-angiogenic properties	MM, AL amyloidosis, MF, Waldenström’s macroglobulinemia, sarcoma, lung cancer, HIV	neutropenia, fatigue, asthenia, anemia, constipation, nausea, diarrhea, dyspnea, upper respiratory tract infections, back pain, pyrexia
	2). Anti-proliferative effects		
	i. Enhances degradation of Ikaros and Aiolos		
	ii. Induces cell cycle arrest		
	3). Immunomodulatory		
	a). Increases number of NK cells and NK cell ADCC		
	b). reverts Th2 cells into Th1 like effector cells		
	c). Suppresses Treg multiplication		
	d). Increases production of IFNγ, IL2 and IL-10		
	e). Co-stimulation of tyrosine phosphorylation		
	f). Activation of PI3 kinase signaling pathway		

Abbreviation: IMiDs, immunomodulatory drugs; PCDs, plasma cell diseases; MM, multiple myeloma; CLE, cutaneous lupus erythematosus; GVHD, Graft-versus-host disease; RA, rheumatoid arthritis; AS, Ankylosing spondylitis; CD, Crohn’s disease; CHF, congestive heart failure; CRC, colorectal cancer; FL, Follicular lymphoma; MCL, mantle cell lymphoma; DBLCL, Diffuse large B-cell lymphoma; CNS, central nervous system; MDS, myelodysplastic syndrome; MF, myelofibrosis; HIV, human immunodeficiency virus; DVT, deep vein thrombosis; AIHA, autoimmune hemolytic anemia; ITP, idiopathic thrombocytopenic purpura.

### 3.1 Antitumor Effect of IMiDs in MM

The deletion of Ikaros and Aiolos maintains the occurrence and progress of MM by contributing to downregulate c-Myc and IRF4 (Bjorklund et al., 2015). There is a positive feedback loop between IRF4 and c-Myc; IRF4 combines with the region of c-myc promoter and promotes c-myc expression, while the C-Myc protein transactivates the IRF4 gene directly (Shaffer et al., 2008). The target genes of C-Myc cover genes that regulate cell metabolism (GLUT1), cell cycle (CDKs, cyclins, and E2FTF), and apoptosis (Jovanović et al., 2018). On the side, Krüppel-like factor 2 (KLF2) and B lymphocyte maturation inducing protein-1 (Blimp-1) exert a significant effect on regulating the growth and survival of MM. In the positive feedback loop, IRF4 and KLF2 transactivate and promote the expression of each other and are also upregulated by KDM3A via removing H3K9me marks at promoters (Ohguchi et al., 2016). Therefore, the lessening of each protein induced by IMiDs represses the expression of integrin alpha-4/beta-7, thus affecting the migration and homing of MM cells to bone marrow. In addition, the expression and activity of Blimp-1 decreased by IMiDs through a variety of mechanisms: 1) Blimp-1 is repressed at the transcriptional level as the target gene of IRF4; 2) Aiolos interacts with Blimp-1 and synergistically inhibits apoptosis-related genes; and 3) ubiquitination and proteasome degradation of Blimp-1 can be enhanced directly (Hung et al., 2016). Moreover, the degradation of Ikaros and Aiolos induced by IMiDs can upregulate the surface expression of CD38 in MM cells, preparing for the antibody-dependent cytotoxicity induced by daratumab in NK cells (Fedele et al., 2018).

### 3.2 Immunomodulatory Activity of IMiDs in MM

IKZF TFs are central to regulate the innate or adaptive immune response in patients with MM. The ILCs consist of lymphoid tissue inducer cells, NK cells, ILC1s, ILC2s, and ILC3s (Vivier et al., 2018; Krabbendam et al., 2021). For NK cells, their activity induced by IMids is due to the fact that T lymphocytes are stimulated to produce IL-2 (Gandhi et al., 2014). Drugs also directly bind to activate kinase ZAP70, downregulate Aiolos, and enhance the expression of cytotoxic molecular granzyme B (Hideshima et al., 2021). Len directly enhances the activity of NK cells by increasing the proportion of synapses that can penetrate IFN-γ vesicles and increasing the production of IFN-γ (Lagrue et al., 2015). Len and pomalidomide increased the MICA expression of NK cell-mediated immune surveillance molecule NKG2D ligand and PVR/CD155 of dNaM-1 ligand on the human MM cell membrane, thus enhancing the sensitivity to the identification and killing of MM cells (Fionda et al., 2015). As for ILCs, it was demonstrated that the activity of ILCs was enhanced by reducing Ikaros and Aiolos protein (Bald et al., 2019). The 2 TFs are significant regulators for the conversion of ILC3 into ILC1/NK cells. Len increased the expression of certain ILC3-related genes (such as rorc, baff, il22, and nrp1) and upregulated the proportion of ILC3 producing IL-22. Concomitantly, a process of transdifferentiation of ILC3 to ILC1 was inhibited by Len with the downregulation of Aiolos and depression of ILC1-associated transcripts (prf1, gzmb, cd244, lef1, and ncr3). After treatment with Len, DC differentiated from peripheral blood and bone marrow monocytes in patients with MM expressed higher standards of cytokines and chemokines (IL-8, TNF, CCL2, and CCL5) and strengthened the function to stimulate the proliferation of allogeneic T cells (Costa et al., 2017).

IMiDs promote specific CD4^+^ and CD8^+^ T cell responses in MM, whereas they inhibit the expansion and function of Tregs. T cells with less expression of Ikaros require less TCR to participate in immune cell activation, show greater proliferation response to IL-2, and are less sensitive to inhibitors of TCR and IL-2R signal transduction, suggesting that the lower the expression of Ikaros, the lower the T cell activation threshold (Avitahl et al., 1999). Besides, the loss of IKZFs is the reason for the increase of IFN-γ, IL-21, and IL-2 production by T cells treated with IMiDs (Gandhi et al., 2014; Brissot et al., 2015). In addition, Len also decreased the levels of Ikaros and Aiolos in chimeric antigen receptor (CAR) T-cells, which helped to enhance the ability of CAR T-cells to restore IL-2 gene transcription and facilitate IL-2 secretion against solid cancer cells (Wang et al., 2020b).

## 4 Clinical Applications

### 4.1 Immunomodulatory Drugs

IMiDs, including thalidomide, Len, and pomalidomide, are effective in treating certain hematological malignancies, in combination with steroids, proteasome inhibitors (PIs), or monoclonal antibodies, such as MM (Gao et al., 2020), MDS with deletion of chromosome 5q (Fenaux et al., 2011), MCL (Ruan et al., 2018), and CLL (Vitale et al., 2016). IMiDs exert their antitumor activity directly through different treatment-related effects, such as inhibiting angiogenesis, repressing cancer cell proliferation, and inducing apoptosis (Le Roy et al., 2018). The cellular target of IMiDs is CRBN, a ubiquitous protein that acts as a substrate receptor for Cullin-4-RING E3 ubiquitin ligase complexes, as well as DDB1, ROC1, and CUL4.

#### 4.1.1 Treatment of IMiDs in MM

Different IMiDs have been applied to different conditions. Thalidomide was the first IMiD to be discovered, and its history served as a lesson in the history of drug development (Franks et al., 2004). Len is commonly used in new MM patients, maintenance therapy after transplant, and relapsed/refractory MM (RRMM) patients, while pomalidomide is only applied to RRMM (Zou et al., 2020).

Patients with newly diagnosed MM (NDMM) who were unsuitable for stem cell transplantation are associated with a considerable benefit in progression-free survival (PFS) [lenalidomide-dexamethasone vs. melphalan-prednisone-thalidomide (MPT); HR, 0.72; *p* < 0.001] and overall survival (OS) at the interim analysis and less frequent grade 3 or 4 toxic and adverse events (70 vs. 78%), with continuous lenalidomide-dexamethasone intervention until disease progression compared with MPT (NCT00689936) (Benboubker et al., 2014). For patients with MM after transplantation, maintenance therapy with Len significantly extended PFS (41 months, vs. 23 months with placebo; HR, 0.50; *p* < 0.001), while differences in OS were not discovered in two cohorts (NCT00430365) (Attal et al., 2012). Identically, Len maintenance significantly improved PFS in patients with NDMM but did not enhance OS in the trial population (ISRCTN49407852) (Jackson et al., 2019). Early intervention with Len in smoldering MM notably decelerates progression to symptomatic MM and the damage of target-organ. The 1-, 2-, and 3-years progression-free survival for the lenalidomide and observation arm was 98 vs. 89%, 93 vs. 76%, and 91 vs. 66%, respectively (NCT01169337) (Lonial et al., 2020). Moreover, in contrast to high-dose dexamethasone, pomalidomide plus low-dose dexamethasone significantly prolonged median PFS (4.0 months, vs. 1.9 months with high-dose dexamethasone; HR, 0.48; CI, 0.39–0.60; *p* < 0.0001), and is considered a new medical strategy for patients with RRMM, despite the fact that the occurrence of grades 3–4 neutropenia were higher (NCT01311687) (Miguel et al., 2013). In another phase III trial, on the basis of BTZ and dexamethasone, pomalidomide was added in the treatment of patients with RRMM to significantly improve PFS (11.20 vs. 7.10 months; HR, 0.61; 95%CI, 0·49–0.77; *p* < 0·0001), with acceptable hemocyte toxicity (NCT01734928) (Richardson et al., 2019). In addition to malignancy, pomalidomide is a well-tolerated and efficient treatment for advanced steroid-refractory chronic graft-versus-host disease (cGVHD), indicating the antifibrotic effects of pomalidomide are related to the increased levels of blood regulatory T cells and IL-2 (NCT01688466) (Curtis et al., 2021).

Subcutaneous BTZ delivery + lenalidomide + dexamethasone (VRD) is a safe and effective therapeutic schedule for induction in NDMM with valuable partial responses (55.6% by cycle 3, 63.8% by cycle 4, 68.3% by cycle 5, and 70.4% after induction) (NCT01916252) (Rosiñol et al., 2019). Furthermore, pretreatment of cells with BTZ, a PI, leads to the accumulation of Ikaros, thus suppressing the efficacy of Len (Shi et al., 2015). Nevertheless, with the therapy of Len combined with BTZ, Ikaros was demonstrated to be degraded by a pathway independent from the autophagy and proteasome pathways, such as activating calpain and caspase to downregulate Ikaros by calcium flux (Ganesan et al., 2020).

Pan-proviral insertion in murine malignancy kinase inhibitors covering SGI1776 and LGH447 presented inspiriting results in patients with RRMM, which was associated with upregulation of CRBN and downregulation of Ikaros and Aiolos (Zheng et al., 2019). Nonselective HDAC inhibitors like A452 induce synergistic cytotoxicity of MM without modifying CRBN expression combined with IMiDs, thus downregulating IKZF1/3, c-Myc, and IRF4 (Won et al., 2019).

#### 4.1.2 Treatment of IMiDs in Leukemia

IMiDs are conventionally used for the therapy of MM, MDS, and B-cell lymphoma; nevertheless, little is realized about the efficacy of IMiDs in AML (Le Roy et al., 2018) (Table 3). Len induces degradation of Ikaros, reducing the expression of GPR68 and RCAN1, which upregulates the Ca2+/calpain pro-apoptotic pathway and inhibits the CaN pro-survival pathway, respectively. Hence, cyclosporine, a CaN inhibitor, is able to extend the therapeutic potential of Len to MDS as well as AML without affecting immune function (Dou and Fang, 2020). Compared to azacytidine (AZA) monotherapy, patients with higher-risk MDS treated with AZA in conjunction with Len or Vorinostat achieved an overall response rate (ORR). In stratified analysis, patients with CMML could benefit from AZA plus Len (NCT01522976) (Sekeres et al., 2017). In patients with 5q-deletion-related MDS, Len rendered significant clinical outcomes compared with thalidomide and pomalidomide, which was related to induced degradation of CK1 *α* (Krönke et al., 2015; Petzold et al., 2016). Len was revealed to be given safely in patients with a relapsed AML/MDS post-allograft combined with AZA without reversing impaired INF-γ/TNF-α production; 7 of 15 (47%) patients ultimately reached a major clinical response after LEN/AZA therapy (ISCRCTN98163167) (Craddock et al., 2019). For patients with AML, the results of the current study illustrated that the addition of Len to standard remission induction chemotherapy (cytarabine + daunorubicin) could not ameliorate the therapeutic effect of elderly patients with AML (NTR2294) (Ossenkoppele et al., 2020).

**TABLE 3:** Ongoing clinical trials of IMiDs in tumors.

NCT number	Phase	Status	Condition	Intervention
**Lenalidomide (also known as Revlimid, CC-5013, and CDC-501)**				
NCT01996865	III	Active, not recruiting	Non Hodgkin Lymphoma	Lenalidomide + Rituximab
NCT04038411	IV	Recruiting	NK/T Cell Lymphoma	PD-1 Antibody + Chidamide + Lenalidomide + Etoposide
NCT03829371	IV	Recruiting	MM	Velcade + Melphalan + Prednisone + Lenalidomide + Dexamethasone
NCT03901963	III	Recruiting	MM	Daratumumab + Lenalidomide
NCT01938001	III	Active, not recruiting	Lymphoma, Non-Hodgkin	Rituximab + Lenalidomide
NCT02659293	III	Active, not recruiting	MM	Lenalidomide + Carfilzomib + Dexamethasone
NCT04217967	IV	Recruiting	MM	Ixazomib + Lenalidomide
NCT01090089	III	Active, not recruiting	MM	Lenalidomide + Dexamethasone + PBSCT
NCT04490707	III	Recruiting	Acute Myeloid Leukemia in Remission	Azacitidine + Lenalidomide
NCT03952091	III	Recruiting	Relapse/Refractory MM	TJ202 + Lenalidomide + Dexamethasone
NCT04040491	III	Recruiting	Peripheral T-cell Lymphoma	PD-1 blocking antibody + chidamide + Lenalidomide + Gemcitabine
NCT04071457	III	Recruiting	MM	Lenalidomide + Daratumumab
NCT00843882	III	Active, not recruiting	Chronic Myelomonocytic Leukemia	Epoetin Alfa + Lenalidomide
NCT02076009	III	Active, not recruiting	MM	Daratumumab + Lenalidomide + Dexamethasone
NCT02215980	III	Active, not recruiting	MM	Lenalidomide + Dexamethasone
NCT03836014	III	Recruiting	Relapse MM	Daratumumab + Lenalidomide + Dexamethasone
NCT04270409	III	Recruiting	Plasma Cell Myeloma	Isatuximab + Lenalidomide + Dexamethasone
NCT03652064	III	Active, not recruiting	MM	Daratumumab + Bortezomib + Lenalidomide + Dexamethasone
NCT01476787	III	Active, not recruiting	Follicular Lymphoma	Lenalidomide + R-CHOP + CVP + Bendamustine
NCT03729804	III	Recruiting	MM	Carfilzomib + Lenalidomide + Dexamethasone + Bortezomib
NCT01650701	III	Active, not recruiting	Follicular Lymphoma	Lenalidomide + R-CHOP + CVP + Bendamustine
NCT02252172	III	Active, not recruiting	MM	Daratumumab + Lenalidomide + Dexamethasone
NCT01685814	III	Active, not recruiting	Previously Untreated Symptomatic MM	Lenalidomide + Bortezomib + ASCT + allo-HSCT
NCT04824092	III	Recruiting	Diffuse Large B-cell Lymphoma	Tafasitamab + Lenalidomide + Rituximab + Cyclophosphamide + Doxorubicin + Vincristine + Prednisone
NCT01564537	III	Active, not recruiting	Relapsed/Refractory MM	Ixazomib + Lenalidomide + + Dexamethasone
NCT02390869	III	Recruiting	Lymphoma, Follicular	Rituximab + Lenalidomide
NCT04680052	III	Recruiting	Follicular Lymphoma, Marginal Zone Lymphoma	Tafasitamab + Rituximab + Lenalidomide
NCT01208662	III	Active, not recruiting	MM	Lenalidomide + Bortezomib + Dexamethasone + ASCT
NCT01093196	III	Active, not recruiting	MM	Melphalan + Prednisone + Lenalidomide + Cyclophosphamide + Dexamethasone
NCT03937635	III	Recruiting	Smoldering Plasma Cell Myeloma	Daratumumab + Dexamethasone + Lenalidomide
NCT03617731	III	Active, not recruiting	MM	Lenalidomide + Bortezomib Dexamethasone + Isatuximab
NCT04751877	III	Not yet recruiting	MM	Isatuximab + Lenalidomide + Bortezomib + Dexamethasone
NCT03173092	III	Recruiting	MM	Ixazomib + Lenalidomide + Dexamethasone
NCT03710603	III	Active, not recruiting	MM	Daratumumab + Velcade + Lenalidomide + Dexamethasone
NCT03319667	III	Active, not recruiting	Plasma Cell Myeloma	Isatuximab + Bortezomib + Lenalidomide + Dexamethasone + Acetaminophen + Ranitidine + Diphenhydramine
NCT00644228	III	Active, not recruiting	Stage I-III Plasma Cell Myeloma	Bortezomib + Dexamethasone + Lenalidomide
NCT04712097	III	Not yet recruiting	Relapsed/Refractory Follicular Lymphoma	Mosunetuzumab + Lenalidomide + Rituximab + Tociluzumab
NCT02495922	III	Active, not recruiting	MM	Elotuzumab + Lenalidomide + Bortezomib + Dexamethasone
NCT03948035	III	Recruiting	Newly Diagnosed MM	Elotuzumab + Carfilzomib + Lenalidomide + Dexamethasone + ASCT
NCT01865110	III	Active, not recruiting	Mantle Cell Lymphoma	R-CHOP + R-HAD + Lenalidomide
NCT01850524	III	Active, not recruiting	MM	Ixazomib + Dexamethasone + Lenalidomide
NCT00551928	III	Active, not recruiting	Newly Diagnosed MM	Melphalan + Lenalidomide + Prednisone
NCT03859427	III	Recruiting	Relapsed/Refractory MM	Carfilzomib + Lenalidomide + Dexamethasone
NCT03941860	III	Recruiting	Plasma Cell Myeloma	Ixazomib Citrate + Lenalidomide
NCT04404283	III	Recruiting	Diffuse Large B-cell Lymphoma	Brentuximab vedotin + Rituximab + Lenalidomide
NCT01091831	III	Active, not recruiting	MM	Cyclophosphamide + Lenalidomide + Dexamethasone + Melphalan
NCT03720041	III	Recruiting	MM	Ixazomib + Lenalidomide + Dexamethasone
NCT00602641	III	Active, not recruiting	Plasma Cell Myeloma	Lenalidomide + Melphalan + Prednisone + Thalidomide
NCT01863550	III	Active, not recruiting	Plasma Cell Myeloma	Bortezomib + Carfilzomib + Dexamethasone + Lenalidomide
NCT01335399	III	Active, not recruiting	MM	Lenalidomide + Dexamethasone + Elotuzumab
NCT02285062	III	Active, not recruiting	Lymphoma, Large B-Cell, Diffuse	Lenalidomide + Rituximab + Cyclophosphamide + Doxorubicin + Prednisone + Vincristine
NCT01208766	III	Active, not recruiting	MM	Bortezomib + Melphalan + Prednisone + Lenalidomide + Dexamethasone
NCT04923893	III	Not yet recruiting	MM	Bortezomib + Dexamethasone + Lenalidomide + Cilta-cel + Cyclophosphamide + Fludarabine
NCT00098475	III	Active, not recruiting	DS Stage I-III Plasma Cell Myeloma	Dexamethasone + Lenalidomide + Thalidomide
NCT00114101	III	Active, not recruiting	DS Stage I-III Plasma Cell Myeloma, Refractory Plasma Cell Myeloma, Smoldering Plasma Cell Myeloma	Autologous Hematopoietic Stem Cell Transplantation + Lenalidomide + Melphalan + PBSCT
NCT02544308	III	Active, not recruiting	Plasmacytoma	Lenalidomide + Dexamethasone
NCT02516696	III	Active, not recruiting	MM	Clarithromycin + Lenalidomide + Dexamethasone
NCT04834024	III	Not yet recruiting	Follicular Lymphoma, Marginal Zone Lymphoma	Recombinant Humanized Monoclonal Antibody MIL62 + Lenalinomide
NCT04152577	III	Recruiting	Lymphoma, B-Cell	R-DA-EPOCH + R-CHOP + R-HD MTX
NCT02516423	III	Active, not recruiting	Solitary Osseous Plasmacytoma	Ixazomib + Lenalidomide + Dexamethasone + Zoledronic acid
NCT04483739	III	Recruiting	MM	Carfilzomib + Lenalidomide + Dexamethasone + Isatuximab
NCT02575144	III	Active, not recruiting	MM	Clarithromycin + Lenalidomide + Dexamethasone
NCT04096066	III	Recruiting	MM/New Diagnosis Tumor	Carfilzomib + Lenalidomide + Dexamethasone
NCT01169337	II/III	Active, not recruiting	Light Chain Deposition Disease, Smoldering Plasma Cell Myeloma	Lenalidomide
NCT03151811	III	Active, not recruiting	MM	Melflufen + Pomalidomide + Dexamethasone
NCT04287660	III	Recruiting	MM	Clarithromycin + Lenalidomide + Dexamethasone + CAR T-cells
NCT04566328	III	Recruiting	Plasma Cell Myeloma, RISS Stage I-II Plasma Cell Myeloma	Bortezomib + Daratumumab + Hyaluronidase-fihj + Dexamethasone + Lenalidomide
NCT04224493	III	Recruiting	Relapsed/Refractory Follicular Lymphoma	Tazemetostat + Lenalidomide + Rituximab
NCT03742297	III	Recruiting	Newly Diagnosed MM	Lenalidomide + Carfilzomib + Bortezomib + Daratumumab + Dexamethasone + Prednisone + Melphalan
NCT03829371	III	Recruiting	MM	Velcade + Melphalan + Prednisone + Lenalidomide + Dexamethasone
NCT03934684	III	Recruiting	Relapsed/Refractory MM	Carfilzomib + Dexamethasone + Lenalidomide
NCT03908138	III	Recruiting	MM	Lenalidomide, Bortezomib + Dexamethasone
NCT04181827	III	Recruiting	MM	JNJ-68284528 + Pomalidomide + Bortezomib + Dexamethasone + Daratumumab
NCT03180736	III	Active, not recruiting	MM	Daratumumab + Pomalidomide + Dexamethasone
NCT04989140	III	Not yet recruiting	MM	Ixazomib + Pomalidomide + Dexamethasone
NCT04934475	III	Not yet recruiting	MM	Isatuximab + ASCT
NCT03428373	II/III	Recruiting	Relapse MM, MM Progression, MM Stage I-III	Lenalidomide + Dexamethasone + Rivaroxaban + Aspirin
NCT04348006	III	Recruiting	Newly Diagnosed MM	Cyclophosphamide + Dexamethasone + Lenalidomide + Bortezomib
NCT03651128	III	Recruiting	MM	bb2121 + Daratumumab + Pomalidomide + Dexamethasone + Bortezomib + Ixazomib + Lenalidomidem Carfilzomib + Elotuzumab
NCT03143049	III	Recruiting	Relapse MM	Pomalidomide + Cyclophosphamide + Dexamethasone
**Thalidomide (also kown as 3-phthalimidoglutarimide, CC-5013, and Thalomid)**				
NCT02507336	II	Active, not recruiting	Mantle Cell Lymphoma	Thalidomide
NCT03143036	II	Recruiting	Relapse/Refractory Myeloma	Daratumumab + Thalidomide + Dexamethasone
NCT03140943	II	Recruiting	Relapsed/Refractory MM	Carfilzomib + Thalidomide + Dexamethasone
NCT04891744	I/II	Not yet recruiting	MM	Selinexor + Thalidomide + Dexamethasone
NCT04382300	II	Recruiting	Non-small-cell Lung Cancer	Pyrotinib + Thalidomide
NCT03062800	II	Recruiting	Advanced NSCLC	Thalidomide + Pemetrexed + Cisplatin + Carboplatin
NCT03896737	II	Recruiting	MM	Daratumumab + Velcade + Cyclophosphamide + Dexamethasone + Thalidomide
NCT02586038	II	Active, not recruiting	MM	MLN9708 + Dexamethasone + Cyclophosphamide + Thalidomide
NCT03143036	II	Recruiting	Relapse/Refractory Myeloma	Daratumumab + Thalidomide + Dexamethasone
NCT00602641	III	Active, not recruiting	Plasma Cell Myeloma	Lenalidomide + Melphalan + Prednisone + Thalidomide
NCT00098475	III	Active, not recruiting	DS Stage I-III Plasma Cell Myeloma	Dexamethasone + Lenalidomide + Thalidomide
NCT01554852	III	Active, not recruiting	MM	Cyclophosphamide + Lenalidomide + Dexamethasone + Thalidomide + Carfilzomib + Protocol + Vorinostat + Melphalan + ASCT
NCT02085655	III	Recruiting	Extranodal NK-T-Cell Lymphoma	Pegaspargase + Gemcitabine + Oxaliplatin + Methotrexate + Dexamethasone + Thalidomide
NCT02541383	III	Active, not recruiting	MM	Bortezomib + Thalidomide + Dexamethasone + Daratumumab
NCT04941937	II	Not yet recruiting	MM	Selinexor + Thalidomide + Lenalidomide + Pomalidomide + Dexamethasone
NCT02891811	II	Recruiting	MM	Carfilzomib + Thalidomide + Lenalidomide + Dexamethasone
NCT01661400	I	Recruiting	Glioma, Neuroectodermal Tumors, Wilms Tumor, Rhabdomyosarcoma, Sarcoma, Ewing, Osteosarcoma, Retinoblastoma	Metronomic Cyclophosphamide + Thalidomide
NCT03792620	III	Recruiting	MM Stage I	Cyclophosphamide + Thalidomide + Dexamethasone + Daratumumab
NCT00572169	III	Active, not recruiting	MM	Velcade + Thalidomide + Dexamethasone + Adriamycin + Cisplatin + Cyclophosphamide + Etoposide
NCT04352205	II	Recruiting	Plasma Cell Myeloma	Bortezomib + Daratumumab + Dexamethasone + Lenalidomide + Thalidomide
NCT03759093	II/III	Not yet recruiting	MM	Bortezomib + Cyclophosphamide + Dexamethasone + Thalidomide
NCT03562169	III	Recruiting	MM	Ixazomib + Thalidomide + Dexamethasone + Conventional ASCT
NCT03980002	II	Recruiting	Chronic Lymphocytic Leukemia	FCR + Ibrutinib + BR + Thalidomide
NCT00871013	II	Active, not recruiting	Myeloma	Melphalan + Velcade + Thalidomide + Dexamethasone + Cisplatin + Adriamycin + Cyclophosphamide + Etoposide
NCT00869232	II	Active, not recruiting	MM	Velcade + Melphalan + Thalidomide + Dexamethasone + Cisplatin + Adriamycin + Cyclophosphamide + Etoposide
NCT03004287	II	Active, not recruiting	MM	Carfilzomib + Thalidomide + Dexamethasone + Daratumumab + Cisplatin + Adriamycin + Cyclophosphamide + Etoposide + Melphalan + ASCT + Lenalidomide + Bortezomib
NCT01356290	II	Recruiting	Medulloblastoma Recurrent, Ependymoma Recurrent, ATRT Recurrent	Bevacizumab + Thalidomide + Celecoxib + Fenofibric acid + Etoposide + Cyclophosphamide + Cytarabine
NCT01998971	I	Active, not recruiting	MM	Daratumumab + Velcade + Pomalidomide + Dexamethasone + Melphalan + Prednisone + Thalidomide + Diphenhydramine + Acetaminophen + Carfilzomib + Lenalidomide + Montelukast
**Pomalidomide (also known as POMALYST, Actimid, CC-4047)**				
NCT04762745	I/II	Not yet recruiting	Relapsed/Refractory MM	Pomalidomide + Bendamustine + Dexamethasone
NCT03257631	II	Active, not recruiting	Medulloblastoma	Pomalidomide
NCT04577755	II	Not yet recruiting	Skin Kaposi Sarcoma	Pomalidomide
NCT03715478	I/II	Recruiting	Relapsed/Refractory MM	Pomalidomide + Dexamethasone
NCT02415153	I	Active, not recruiting	Neurofibromatosis Type 1, Recurrent Childhood Brain Stem Glioma, Recurrent Childhood Visual Pathway Glioma, Recurrent//Refractory Primary Central Nervous System Neoplasm	Pomalidomide
NCT01997840	I/II	Active, not recruiting	MM	ACY-1215 + Pomalidomide + Dexamethasone
NCT02045017	II	Active, not recruiting	MM	Pomalidomide + Dexamethasone
NCT04902443	I	Not yet recruiting	Kaposi Sarcoma, EBV/KSHV-associated Lymphomas	Pomalidomide + Nivolumab
NCT04584307	II	Not yet recruiting	MM	Elotuzumab + Pomalidomide
NCT03798314	I	Active, not recruiting	Recurrent/Refractory Primary Vitreoretinal DLBCL	Nivolumab + Pomalidomide
NCT01734928	III	Active, not recruiting	MM	Pomalidomide + Bortezomib + Dexamethasone
NCT03601806	II	Recruiting	Skin Kaposi Sarcoma	Pomalidomide
NCT02004275	I/II	Active, not recruiting	Relapse MM	Pomalidomide + Ixazomib + Dexamethasone
NCT04176718	II	Recruiting	Relapse/Refractory MM, MM	Daratumumab + Carfilzomib + Pomalidomide + Dexamethasone
NCT01754402	I/II	Active, not recruiting	MM	Bendamustine + Pomalidomide + Dexamethasone
NCT02406222	II	Active, not recruiting	MM	Pomalidomide + Dexamethasone + Cyclophosphamide
NCT03151811	III	Active, not recruiting	MM	Melflufen + Pomalidomide + Dexamethasone
NCT03143985	I	Recruiting	MM	Vactosertib + Pomalidomide
NCT01946477	II	Recruiting	MM	Pomalidomide + Dexamethasone + Daratumumab
NCT03180736	III	Active, not recruiting	MM	Daratumumab + Pomalidomide + Dexamethasone
NCT04850599	II	Not yet recruiting	Recurrent/Refractory Plasma Cell Myeloma	Carfilzomib + Isatuximab + Pomalidomide
NCT04790474	II	Recruiting	Relapse/Refractory MM	Ixazomib + Pomalidomide + Dexamethasone
NCT03030261	II	Recruiting	Relapse MM	Elotuzumab + Pomalidomide + Dexamethasone
NCT04802161	II	Not yet recruiting	Acute Myeloid Leukemia, Chronic Myelomonocytic Leukemia, Myelodysplastic Syndrome	Liposome-encapsulated Daunorubicin-Cytarabine + Pomalidomide
NCT02990338	III	Active, not recruiting	Plasma Cell Myeloma	Isatuximab + Pomalidomide + Dexamethasone
NCT04764942	I/II	Recruiting	Recurrent/Refractory Plasma Cell Myeloma	Carfilzomib + Dexamethasone + Pomalidomide + Selinexor
NCT04094961	I/II	Recruiting	Relapse MM	Ixazomib + Pomalidomide + Dexamethasone
NCT03756896	II	Recruiting	Plasma Cell Myeloma	Carfilzomib + Dexamethasone + Pomalidomide
NCT01166113	I/II	Active, not recruiting	MM	Pomalidomide + Cyclophosphamide + Prednisone
NCT02659930	I	Recruiting	Kaposi Sarcoma	liposomal Doxorubicin + Pomalidomide
NCT04508790	II	Recruiting	Recurrent/Refractory Plasma Cell Myeloma	Dexamethasone + Leflunomide + Pomalidomide
NCT02400242	I	Active, not recruiting	MM	ACY-241 + Pomalidomide + Dexamethasone
NCT03015922	I	Active, not recruiting	MM	Lenalidomide + Pomalidomide + Reolysin
NCT03590652	II	Recruiting	Relapsed/Refractory MM	Ixazomib + Pomalidomide + Dexamethasone + Daratumumab
NCT04162210	III	Recruiting	MM	Belantamab mafodotin + Pomalidomide + Dexamethasone)
NCT04191616	II	Recruiting	Relapsed/Refractory MM	Carfilzomib + Dexamethasone + Pomalidomide
NCT02542657	I/II	Active, not recruiting	Myeloma	Clarithromycin + Dexamethasone + Ixazomib + Pomalidomide
NCT04883242	II	Recruiting	Recurrent/Refractory Plasma Cell Myeloma	Carfilzomib + Dexamethasone + Isatuximab + Pomalidomide
NCT03143049	III	Recruiting	Relapse MM	Pomalidomide + Cyclophosphamide + Dexamethasone
NCT03683277	II	Not yet recruiting	Relapsed/Refractory MM	Ixazomib + Pomalidomide + Dexamethasone
NCT04843579	II	Not yet recruiting	Myeloma/Refractory MM	Selinexor + Clarithromycin + Pomalidomide + Dexamethasone
NCT01665794	I/II	Recruiting	MM	Pomalidomide + Carfilzomib + Dexamethasone + Daratumumab
NCT04661137	II	Recruiting	MM	Selinexor + Carfilzomib + Pomalidomide + Daratumumab + Dexamethasone
NCT03539744	III	Recruiting	MM	Pomalidomide + Dexamethasone + Venetoclax
NCT04700176	II	Not yet recruiting	MM	Daratumumab + Pomalidomide + All-trans retinoic acid + Dexamethasone
NCT04302324	II	Recruiting	Refractory/Relapse MM	Daratumumab + Clarithromycin + Pomalidomide + Dexamethasone
NCT02547662	II	Active, not recruiting	Plasma Cell Leukemia/Plasma Cell Myeloma/Plasmacytoma	Ixazomib citrate + Pomalidomide
NCT02654132	II	Active, not recruiting	MM	Elotuzumab + Pomalidomide + Dexamethasone
NCT04484623	III	Recruiting	MM	Belantamab mafodotin + Pomalidomide + Dexamethasone + Bortezomib
NCT01745588	II	Active, not recruiting	MM	Pomalidomide + Dexamethasone + Clarithromycin
NCT03202628	II	Active, not recruiting	Recurrent/Refractory Plasma Cell Myeloma	ASCT + Dexamethasone + Ixazomib + Pomalidomide
NCT03287908	I	Recruiting	Relapsed/Refractory MM	AMG 701 + Pomalidomide + Dexamethasone
NCT04124497	II	Recruiting	MM/Deletion 17P Syndrome	Daratumumab + Pomalidomide + Dexamethasone
NCT04667663	I	Not yet recruiting	MM	Daratumumab + Cyclophosphamide + Pomalidomide + Dexamethasone
NCT01575925	I	Active, not recruiting	MM with Renal Impairment	Pomalidomide + Dexamethasone
NCT04989140	IV	Not yet recruiting	MM	Ixazomib + Pomalidomide + Dexamethasone
NCT04835129	II	Not yet recruiting	MM	Isatuximab + Pomalidomide + Elotuzumab + Dexamethasone
NCT03841565	II	Recruiting	Recurrent Plasma Cell Myeloma	Daratumumab + Dexamethasone + Pomalidomide
NCT02185820	I/II	Active, not recruiting	MM	Carfilzomib + Pomalidomide + Dexamethasone
NCT03713294	II	Recruiting	Refractory Plasma Cell Myeloma	Dexamethasone + Elotuzumab + Pomalidomide
NCT04181827	III	Recruiting	MM	JNJ-68284528 + Pomalidomide + Bortezomib + Dexamethasone + Daratumumab
NCT04762745	I/II	Not yet recruiting	Relapsed, Refractory, MM	Pomalidomide + Bendamustine + Dexamethasone
NCT03170882	II	Active, not recruiting	Relapsed/Refractory MM	Ixazomib + Pomalidomide + Dexamethasone
NCT02726581	III	Active, not recruiting	MM	Nivolumab + Elotuzumab + Pomalidomide + Dexamethasone
NCT04287855	II	Active, not recruiting	Relapse/Refractory MM	Isatuximab + Carfilzomib + Pomalidomide + Dexamethasone
NCT02188368	II	Active, not recruiting	MM	Pomalidomide + Steroids + Doxorubicin + Carfilzomib + Bortezomib + Clarithromycin + Cyclophosphamide
NCT02616640	I	Active, not recruiting	MM	Durvalumab + Pomalidomide + Dexamethasone
NCT03104270	II	Active, not recruiting	MM	Elotuzumab + Pomalidomide + Carfilzomib + Dexamethasone
NCT03731832	II	Recruiting	Refractory MM	MLN9708 + Pomalidomide + Dexamethasone + Cyclophosphamide
NCT04941937	II	Not yet recruiting	MM	Selinexor + Thalidomide + Lenalidomide + Pomalidomide + Dexamethasone
NCT02939183	I	Active, not recruiting	Relapsed/Refractory MM	Dexamethasone + Pomalidomide
NCT03439280	I/II	Active, not recruiting	Relapsed/Refractory/MM	TAK-079 + Pomalidomide + Dexamethasone
NCT04643002	I/II	Recruiting	Plasma Cell Myeloma Refractory	Isatuximab + Dexamethasone + Pomalidomide + SAR439459 + Belantamab mafodotin
NCT03582033	I	Recruiting	MM	SEA-BCMA + Dexamethasone + Pomalidomide
NCT04108195	I	Recruiting	MM	Daratumumab + Talquetamab + Teclistamab + Pomalidomide
NCT04150965	I/II	Recruiting	Relapsed/Refractory MM	Elotuzumab + Pomalidomide + Dexamethasone + Anti-LAG-3 + Anti-TIGIT
NCT04045795	I	Recruiting	MM	Isatuximab + Pomalidomide + Dexamethasone
NCT02963493	II	Active, not recruiting	MM	Melphalan + Dexamethasone
NCT04942067	I/II	Recruiting	MM	APG2575 + Lenalidomide + Pomalidomide + Dexamethasone
NCT02807454	II	Active, not recruiting	MM	Daratumumab + Durvalumab + Pomalidomide + Dexamethasone
NCT03269136	I	Active, not recruiting	MM	PF-06863135 + Dexamethasone + Lenalidomide + Pomalidomide
NCT02343042	I/II	Recruiting	MM	Selinexor + Dexamethasone + Lenalidomide + Pomalidomide + Bortezomib + Daratumumab + Carfilzomib + Ixazomib + Elotuzumab + Clarithromycin + Belantamab Mafodotin
NCT04895410	I	Not yet recruiting	MM	Lemzoparlimab + Dexamethasone + Carfilzomib + Pomalidomide + Daratumumab
NCT04458831	NA	Recruiting	Plasma Cell Myeloma	Isatuximab + Pomalidomide + Dexamethasone + Carfilzomib
NCT04892446	II	Not yet recruiting	MM	Magrolimab + Daratumumab + Pomalidomide + Dexamethasone + Bortezomib
NCT04925193	II	Not yet recruiting	Relapse MM	Selinexor + Pomalidomide + Daratumumab + Carfilzomib + Dexamethasone
NCT04855136	I/II	Recruiting	MM	BB2121 + CC-220 + BMS-986405 + Pomalidomide + Dexamethasone + Bortezomib
NCT03828292	I	Recruiting	MM	Belantamab mafodotin + Bortezomib + Dexamethasone + Pomalidomide
NCT03732703	I/II	Recruiting	Relapsed/Refractory MM	Abemaciclib + Dexamethasone + Ixazomib + Pomalidomide + Enasidenib + Cobimetinib + Erdafitinib + Venetoclax + Daratumumab + Belantamab mafodotin + Selinexor
NCT04722146	I	Recruiting	MM	Teclistamab + Daratumumab + Pomalidomide + Lenalidomide + Bortezomib + Nirogacestat
NCT03984097	I	Active, not recruiting	MM	TAK-079 + Lenalidomide + Dexamethasone + Bortezomib + Pomalidomide
NCT02294357	II	Active, not recruiting	MM	Carfilzomib + Dexamethasone + Prednisone + Methylprednisolone + Lenalidomide + Pomalidomide
NCT03651128	III	Recruiting	MM	bb2121 + Daratumumab + Pomalidomide + Dexamethasone + Bortezomib + Ixazomib + Lenalidomide + Carfilzomib + Elotuzumab
NCT02206425	I/II	Active, not recruiting	MM	Melphalan + Prednisone + Cyclophosphamide + Dexamethasone + Doxorubicin + Lenalidomide + Pomalidomide
NCT01592370	I/II	Active, not recruiting	Non-Hodgkin’s Lymphoma, Hodgkin Lymphoma, MM	Nivolumab + Ipilimumab + Daratumumab + Pomalidomide + Dexamethasone
NCT01998971	I	Active, not recruiting	MM	Daratumumab + Velcade + Pomalidomide + Dexamethasone + Melphalan + Prednisone + Thalidomide + Diphenhydramine + Acetaminophen + Carfilzomib + Lenalidomide + Montelukast
NCT02719613	II	Active, not recruiting	MM	Elotuzumab + Dexamethasone + Lenalidomide + Bortezomib + Pomalidomide + Nivolumab
NCT03732703	I/II	Recruiting	Relapsed/Refractory MM	Abemaciclib + Dexamethasone + Ixazomib + Pomalidomide + Enasidenib + Cobimetinib + Erdafitinib + Venetoclax + Daratumumab + Belantamab + Selinexor
NCT03269136	I	Active, not recruiting	MM	PF-06863135 + Dexamethasone + Lenalidomide + Pomalidomide
NCT04150965	I/II	Recruiting	Relapsed/Refractory MM	Elotuzumab + Pomalidomide + Dexamethasone
NCT03143985	I	Recruiting	MM	Vactosertib + Pomalidomide
NCT03257631	II	Active, not recruiting	Central Nervous System Neoplasms	Pomalidomide
NCT01734928	III	Active, not recruiting	MM	Pomalidomide + Bortezomib + Dexamethasone
NCT01575925	I	Active, not recruiting	MM	Pomalidomide + Dexamethasone
NCT02188368	II	Active, not recruiting	MM	Pomalidomide + Steroids + Doxorubicin + Carfilzomib + Bortezomib + Clarithromycin + Cyclophosphamide

*Lenalidomide has been widely investigated in clinic, therefore only clinical trials in phase III and IV are included.

Abb: R-CHOP, Rituximab, Cyclophosphamide, Doxorubicin, Vincristine, Prednisone; CVP, Rituximab Cyclophosphamide, Vincristine, Prednisone; ASCT, autologous stem cell transplant; allo-HSCT, allogeneic hematopoietic stem cell transplantation; G-CSF, granulocyte-colony stimulating factor; R-HAD, Rituximab, Cytarabine, Dexamethasone; PBSCT, Peripheral Blood Stem Cell Transplantation; R-HD MTX, Rituximab, Methotrexate; R-DA-EPOCH, Rituximab, Epirubicin, Etoposide, Vincristine, Cyclophosphamide, Prednisone; BR, Rituximab, Bendamustine; VBMCP, Vincristine; BCNU, Cyclophosphamide, Melphalan, Prednisone; VBAD, Vincristine, Adriamycine, Dexamethasone; GDPT, Gemcitabine, Cisplatin,Prednisone, Thalidomide; FCR, Fludarabine, Cyclophosphamide, Rituximab; MM, Multiple myeloma.

In a phase I trial, following timed sequential induction therapy (TST), pomalidomide administration in the early stages of lymphocyte recovery has been revealed to be well tolerated in patients with newly diagnosed AML and adverse cytogenetics of MDS with particularly high CR rates (NCT02029950). The exact mechanism of benefit from pomalidomide application after chemotherapy remains obscure. It is of probability that pomalidomide remarkably decreases Aiolos expression in peripheral blood and bone marrow CD4+/CD8+ T cells, strengthens T cell differentiation and proliferation, and promotes cytokine production (Zeidner et al., 2020). Additionally, pomalidomide may be serviceable for the treatment of HTLV-1 and EBV-induced tumors by causing infected cells to become more vulnerable to innate and adaptive host immune responses (Davis et al., 2019).

Lenalidomide has been approved for application with rituximab in patients with relapsed/refractory follicular lymphoma in the United States. Lenalidomide + rituximab was demonstrated to be a safe and effective chemotherapy-free approach that improves upon single-agent rituximab and may become a substitution to chemoimmunotherapy (Flowers et al., 2020). Efficacy results of rituximab plus lenalidomide were similar to rituximab plus chemotherapy and both regimens were followed by rituximab maintenance therapy among patients with untreated follicular lymphoma. In contrast, the two groups presented different safety profiles. A higher percentage of grades 3 or 4 neutropenia appeared in the rituximab + chemotherapy group (32 vs. 50%) (NCT01476787 and NCT01650701) (Morschhauser et al., 2018). PFS of patients with recurrent indolent lymphoma was notably promoted for lenalidomide plus rituximab compared with rituximab plus placebo (HR, 0.46; 95% CI, 0.34–0.62; *p* < 0.001), and the safety profile of the test group was acceptable (NCT01938001) (Leonard et al., 2019). For patients with DLBCL who achieved CR or PR by R-CHOP induction, a randomized phase III trial demonstrated that Len maintenance monotherapy in elderly patients with DLBCL is safe and effective (mPFS was not reached, vs. 58.9 months with placebo; HR, 0.708; 95% CI, 0.537–0.933; *p* = 0.01) (Thieblemont et al., 2017). Despite inescapable toxicity (63%, vs. 12% in observation group; *p* < 0·0001), Len improved PFS in patients with an MCL post-autograft (NCT02354313) (Ladetto et al., 2021).

Long-term IMiDs used continuously, like maintenance treatment for MM, may raise the frequency of uncontrolled immunostimulatory diseases by inducing a chronic inflammatory response, resulting in certain autoimmune diseases covering vasculitis, rashes, optic neuritis, interstitial pneumonitis, Graves’ disease, and polymyositis (Montefusco et al., 2014; Brissot et al., 2015; Shao et al., 2017; Phan et al., 2020; Shafi et al., 2020; Cippitelli et al., 2021).

### 4.2 Treatment of Cereblon-Modulating Agent

CRBN E3 ligase modulating drugs (CELMoDs), such as avadomide (CC-122), iberdomide (CC-220), CC-885, CC-92480, and novel thalidomide analogs, are in current clinical trials as a monotherapy and in combination (NCT01421524; NCT02773030; NCT03374085) (Table 4). The CELMoDs have strong antitumor and immunostimulatory capabilities in patients with hematological malignancies (Matyskiela et al., 2016; Matyskiela et al., 2018; Ito and Handa, 2019; Rasco et al., 2019; Gao et al., 2020; Hansen et al., 2020).

**TABLE 4:** Clinical trials of CELMoDs in tumors.

Drug	Alias	NCT number	Phase	Conditions	Interventions
CC-122	Avadomide	NCT02509039	1	NHL, Solid Tumors	CC-122
		NCT02859324	1, 2	Unresectable HCC	CC-122, Nivolumab
		NCT02323906	1	HCC	CC-122, Sorafenib
		NCT03834623	2	Advanced Melanoma	CC-122, Nivolumab
		NCT02031419	1	DLBCL, FL	CC-122, CC-223, Rituximab, CC-292
		NCT02417285	1	Relapsed/Refractory DLBCL, iNHL	Obinutuzumab, CC-122
		NCT02406742	1,2	CLL, SLL	CC-122, Ibrutinib, Obinutuzumab
		NCT03283202	1	DLBCL	CC-122, R-CHOP
		NCT01421524	1	NHL, Solid Tumors, MM	CC-122
CC-220	Iberdomide	NCT04882163	1, 2	B-cell Lymphoma	CC-220, Polatuzumab vedotin, Rituximab, Tafasitamab, Gem, DDP, DEX, Bendamustine, Len
		NCT03161483	2	SLE	CC-220, Placebo
		NCT02185040	2	SLE	CC-220, Placebo
		NCT04884035	1	a-BCL	CC-220, R-CHOP, CC-99282
		NCT04464798	1	RR Lymphoma	CC-220, Rituximab, Obinutuzumab
		NCT02773030	1, 2	MM	CC-220, DEX, DARA, BTZ, CFZ
		NCT02192489	2	Skin Sarcoidosis	CC-220, Placebo
		NCT04564703	2	MM	CC-220
		NCT04392037	2	MM	CC-220, cyclophosphamide, DEX
		NCT04855136	1, 2	MM	BB2121, CC-220, BMS-986405, Pom, DEX, BTZ
		NCT03310619	1, 2	NHL, DLBCL, FL	JCAR017, Durvalumab, CC-122, Ibrutinib, CC-220, Relatlimab, Nivolumab, CC-99282
CC-92480	—	NCT03989414	1, 2	MM	CC-92480, BTZ, DEX, DARA, CFZ, Elotuzumab, Isatuximab
		NCT03374085	1, 2	MM	CC-92480, DEX

Abbreviation: NHL, Non-Hodgkin’s lymphoma; HCC, hepatocellular carcinoma; DBLCL, Diffuse large B-cell lymphoma; iNHL, indolent NHL; CLL, Chronic lymphocytic leukemia; FL, Follicular lymphoma; MM, multiple myeloma; BCL, B-cell Lymphoma; SLE, systemic lupus erythematosus; RR, Lymphoma, relapsed/refractory lymphoma; R-CHOP (Rituximab, cyclophosphamide, doxorubicin, vincristine, prednisone); DEX, dexamethasone; DARA, daratumumab; BTZ, bortezomib; CFZ, carfilzomib; Len, Lenalidomide; Gem, gemcitabine; DDP, Cisplatin; Pom, Pomalidomide.

CC-122 (avadomide) was combined with CRL4^CRBN^ E3 ligase to conduce the degradation of Ikaros and Aiolos in MM cells and DLBCL cells (Hagner et al., 2015). CC-122 has been applied to a variety of clinical trials for diverse diseases, including non-Hodgkin’s lymphoma, MM, HCC, melanoma, and CLL/SLL (Rasco et al., 2019). It was found in research in pre-clinical experiments and a phase I clinical trial that avadomide + obinutuzumab was a tolerable therapeutic regimen for plenty of patients with manageable toxicity. Nevertheless, the predetermined activity threshold was not reached in this trial. There is no denying that cereblon-modulators plus anti-CD20 antibodies should be investigated further as a chemotherapy-free approach for relapsed or refractory non-Hodgkin lymphoma (NCT02417285) (Michot et al., 2020). In another phase I clinical trial, avadomide monotherapy presented manageable toxicity and promising pharmacokinetics in patients with solid tumors, NHL, and multiple myeloma. Of five patients with NHL, one realized CR, and two achieved PR (NCT01421524) (Rasco et al., 2019).

CC-220 (iberdomide) is another analog of thalidomide, which enhances the efficacy to downregulate Ikaros and Aiolos by tightly binding to CRL4^CRBN^ E3 ligase for the treatment of RRMM and SLE (Matyskiela et al., 2018). The peripheral blood mononuclear cells of patients with SLE showed strikingly higher levels of IKZF1 (2.1-fold) and IKZF3 (4.1-fold) mRNA than a healthy person. And iberdomide significantly decreased levels of Ikaros and Aiolos protein in B cells, T cells, and monocytes, supporting its further clinical development for treating SLE (NCT01733875) (Schafer et al., 2018).

In addition to the pharmacologic function of CC-220, CC-885 can also induce CRL4^CRBN^-dependent delegation of translation termination factor GSPT1, which cannot be degraded by neither Len nor pomalidomide, demonstrating the spectrum of the different substrates of CC-885 from pomalidomide and Len (Matyskiela et al., 2016). Hence, CC-885 may have the extra potential for AML therapy. Relevant pre-clinical and clinical trials are ongoing and being analyzed due to their novelty. CC-92480 is another novel protein degrader acting on the refractory/relapsed MM (RRMM) of Len (Hansen et al., 2020).

### 4.3 Treatment of Proteasome Inhibitors

PIs are a class of important drugs for the treatment of MM and MCL, and are being studied for the treatment of other diseases. BTZ is the first PI to be approved by the Food and Drug Administration (FDA) of the United States. Carfilzomib and ixazomib have been approved successively, and more drugs are in development (Fricker, 2020). The phase III GIMEMA-MMY-3006 trial illuminated the superiority of the BTZ + thalidomide + dexamethasone (VTD) regimen over thalidomide and dexamethasone (TD) in improving CR and prolonging PFS (HR, 0·60; 95% CI, 0·48–0.76; *p* < 0·0001) as an induction regimen before and intensification therapy after double autologous hematopoietic stem-cell transplantation (ASCT) for patients with NDMM (NCT01134484) (Cavo et al., 2010; Tacchetti et al., 2020). The randomized phase III trial SWOG S0777 and long-term follow-up of SWOG S0777 revealed a similar pattern that the addition of BTZ to Len and Dex for induction therapy led to a statistically and clinically meaningful increase in PFS as well as OS (NCT00644228) (Durie et al., 2017; Durie et al., 2020). Another trial demonstrated that the first single-agent ixazomib option in patients with NDMM not undergoing ASCT promoted mPFS (17.4 vs. 9.4 months; HR, 0.659; 95% CI, 0.542–0.801; *p* < 0.001; median follow-up, 21.1 months) without unexpected toxicity (NCT02312258) (Dimopoulos et al., 2020a). For combination therapy of ixazomib, the addition of ixazomib to a Len and Dex regimen was correlated to longer PFS (20.6 vs. 14.7 months in Len and Dex; HR, 0.74; *p* = 0.01) with limited toxic effects (NCT01564537) (Moreau et al., 2016). Besides, in patients with RRMM, carfilzomib in conjunction with Len and Dex (KRd) generated markedly improved mPFS (26.3 months, vs. 17.6 months in the Len and Dex group; HR, 0.69; 95% CI, 0.57–0.83; *p* = 0.0001) at the interim analysis and presented an improving risk-benefit profile (NCT01080391) (Stewart et al., 2015). However, the KRd scheme cannot effectively improve PFS (34·6 months vs. 34·4 months in the Len and Dex group; HR, 1·04; 95% CI, 0.83–1.31; *p* = 0·74) compared with a control group in patients with NDMM, and had more toxicity (NCT01863550) (Kumar et al., 2020).

### 4.4 Immunotherapy

Immunotherapy is one of the most promising treatments for several cancers. In future clinical trials, early identification of high-risk patients with ALL is essential for the best treatment, and to ensure innovative treatments, such as immunotherapy, are introduced in a timely manner (Stanulla et al., 2018). In some studies about certain solid tumors that lack IKZF1 expression, overexpression of Ikaros leads to increased immune recruitment infiltration and tumor sensitivity to cytotoxic T lymphocyte-associated antigen-4 (CTLA4) and programmed cell death protein 1 (PD1) inhibitors (Chen et al., 2015; Chen et al., 2018). Nevertheless, meaningful MM regression was not achieved by single-drug PD-1 blockade in early clinical studies. Besides, the FDA established that the risks of the pembrolizumab plus pomalidomide and dexamethasone combination outweighed its benefits for patients with RRMM and halted the study (NCT02576977) (Mateos et al., 2019). Equally, the interim analysis results presented that the benefit-risk event of pembrolizumab plus lenalidomide and dexamethasone is adverse for patients with NDMM. Survival and long-term safety follow-up are ongoing (NCT02579863) (Usmani et al., 2019). CAR-T immunotherapy faces many problems in the treatment of solid tumors, and T-cell dysfunction or failure is one of them. Len improved CAR-T function by inducing the degradation of Ikaros and Aiolos, promoting the killing of CD133-CAR-T and increasing the secretion of the cytokine and the proliferation of CD133-CAR-T (Wang et al., 2020b).

### 4.5 Targeted Therapy

Adding imatinib to the therapy for patients with BCR-ABL1 fusion to strengthen the treatment of IKZF1-del patients can eliminate the adverse effect of IKZF1-del in contemporary ALL treatment (Yeoh et al., 2018). Imatinib, as an intensifying therapy for childhood B-ALL with IKZF1-del, significantly decreased the risk of recurrence and improved the 5-years OS from 69.6% in MS2003 to 91.6% in MS 2010 (*p* = 0.007) (NCT0289464) (Yeoh et al., 2018). Besides, ibrutinib also presented enduring single-agent efficacy in patients with relapsed or refractory MCL. A CR rate of 21% and a PR rate of 47% were detected. And the most common adverse events related to treatment were diarrhea, fatigue, and nausea (NCT01236391) (Wang et al., 2013).

DD-03–171 is an optimized Bruton tyrosine kinase (BTK) inhibitor analog, which showed an enhanced anti-proliferation outcome on MCL cells *in vitro* through the degradation of BTK, Ikaros, and Aiolos (Dobrovolsky et al., 2019). Acalabrutinib, a selective second-generation BTK inhibitor, provided superior durable responses and an acceptable toxicity profile in patients with relapsed or refractory MCL. At the median follow-up of 15.2 months, 81% of patients attained an overall response, and 40% of patients achieved a CR. The most common adverse events were headache (38%), diarrhea (31%), fatigue (27%), and myalgia (21%) (NCT02213926) (Wang et al., 2018).

The CK2 inhibitor CX4945 can restore Ikaros function and exert the antileukemia effect *in vitro* or in pre-clinical leukemia models (Gowda et al., 2017b; Borgo et al., 2021). There are also many cancer clinical trials currently running (NCT01199718, NCT00891280, NCT03897036, NCT02128282, NCT03904862, and NCT03571438).

Bromodomain-containing protein 4 (BRD4) is a chromatin-binding protein that was considered to immediately regulate a range of genes involved in the BCR signal pathway, covering Aiolos, B-cell linker, PAX5, and several oncogenes, like MYB. Thalidomide derivatives to various Brd4 small molecule inhibitors through different junctions were used to synthesize CRL4^CRBN^ E3-based PROTAC targeting Brd4. For example, the synthesis of many pre-clinical trials like dBET1, ARV-825, BETd-246, BETd-260, and QCA570. Of course, there are other degradation pathways, such as the CRL2^VHL^ E3-based pathway (Duan et al., 2018). BRD4 inhibitors were suggested to enhance the anti-MCL effect of Len or ibrutinib synergistically. It works even in BTZ-resistant MCL cells (Tsukamoto et al., 2020).

In breast cancer, the application of ginseng polysaccharide was able to suppress MDA-MB-231 cell proliferation by the activation of IKZF1 (Zhou et al., 2020). The anticancer capability of imatinib in Ik6+ and Ph + ALL can be strengthened by plant elements such as Huaier extract (Qu et al., 2019). The use of caffeic acid phenethyl ester analogs in the so-called Achilles heel of myeloma, aimed at the Ikaros/IRF4 axis, showed positive clinical outcomes (Murugesan et al., 2020).

Daratumumab, a monoclonal antibody targeting CD38, has presented substantial efficacy as a monotherapy in heavily pretreated and refractory patients with MM and in conjunction with bortezomib in patients with NDMM as well as with IMiD in patients with RRMM. Overall responses were noted in 29.2% of refractory patients with MM treated with daratumumab monotherapy (NCT01985126) (Lonial et al., 2016; Usmani et al., 2020). In addition, the combined administration may be an ideal backbone for the future research of anti-CD38 monoclonal antibodies in terms of their good safety and oral administration. The addition of daratumumab with Len-Dex induced a high ORR and PFS, markedly decreasing the risk for progression and associated death in patients with RRMM and NDMM who were ineligible for ASCT in contrast to Len-Dex therapy (Dimopoulos et al., 2016). And after 44.3 months of follow-up, triple therapy significantly prolonged PFS in the treated population (44.5 vs. 17.5 months; HR, 0.44; 95% CI, 0.35–0.55; *p* < 0.0001). In terms of adverse profiles, a higher frequency of neutropenia and pneumonia was noticed in the daratumumab group (NCT02076009, NCT02252172) (Facon et al., 2019; Bahlis et al., 2020). Moreover, daratumumab plus Pom-Dex could also reduce the risk of disease progression and improve mPFS (12·4 vs. 6·9 months; HR, 0·63; 95% CI, 0·47–0.85) in patients with RRMM compared to Pom-Dex, and can be considered a new therapeutic strategy (NCT03180736) (Chari et al., 2017; Dimopoulos et al., 2021a). Besides, daratumumab applied with carfilzomib and Dex in patients with RRMM was associated with a promising benefit-risk profile and prolonging PFS (NCT03158688) (Dimopoulos et al., 2020b). In quadruple therapy, induction and remarkable consolidation treatment of daratumumab plus Len, BTZ, and Dex improved response depth and PFS with acceptable safety in patients with transplant-eligible NDMM (NCT02874742, NCT02541383) (Moreau et al., 2019; Voorhees et al., 2020). Isatuximab, another monoclonal antibody targeting CD38, has been evaluated as a monotherapy and combined with Dex in patients with RRMM. The combination of isatuximab and Dex promoted ORR (43.6 vs. 23.9% in the Isa arm) and survival outcomes with no adverse effect on safety (NCT01084252) (Dimopoulos et al., 2021b). On the basis of Pom-Dex, the addition of isatuximab was able to importantly improve PFS in patients with RRMM, particularly for patients who are refractory to Len and a PI (NCT02990338) (Attal et al., 2019).

In phase Ib-III clinical studies, elotuzumab, an immunostimulatory antibody against signaling lymphocytic activation molecule F7 (SLAMF7), achieved a reduction of 30% in the risk of disease progression or death in patients with RRMM combined with Len and Dex (NCT01239797) (Lonial et al., 2015; Dimopoulos et al., 2020c). Particularly among patients with MM who failed after treatment with Len and a PI, the addition of elotuzumab to Pom-Dex tremendously lowered the risk of disease progression or death and prolonged mPFS (10.3 vs. 4.7 months in the control group) versus Pom-Dex alone (NCT02654132) (Dimopoulos et al., 2018). Nevertheless, in the SWOG-1211 trial, elotuzumab combined with BTZ, Len, and Dex did not improve clinical outcomes of patients with NDMM as induction and maintenance treatments (Usmani et al., 2021) (NCT01668719).

### 4.6 Reversal of Drug Resistance

The majority of newly diagnosed patients respond to therapy of an immunomodulator, and yet most develop resistance to administered therapies eventually. Strategies to surmount resistance have been proposed (Mogollón et al., 2019). In MM, distinguishing mechanisms of resistance to both Len and Pom are apparent, therefore, tumors with acquired resistance to pomalidomide react to Len and vice versa (Ocio et al., 2015). STAT3 and MEK1/2 inhibitors have been testified to overcome IMiDs resistance effectively in pre-clinical studies (Ocio et al., 2015; Zhu et al., 2019). Enhancing degradation of CRBN has also been proved to be more sensitive to IMiDs (Kim et al., 2020). Protecting IKZFs from degradation through RUNXs inhibition resulted in conquering the resistance of IMiDs in MM (Zhou et al., 2019). In addition, a synthesized HDAC6 selective inhibitor, A452, is a strategy to overcome resistance to IMiDs (He et al., 2020). Furthermore, Cys reinforced the sensitivity of MDS/AML cells to Len without affecting the activation of T cells (He et al., 2020). It has been reported that the addition of cyclophosphamide to an IMiD could enhance the efficacy of IMiDs, prolong PFS and OS, and even save patients with Len-refractory disease, regardless of whether they have pre-accepted ASCT (NCT02244125) (Garderet et al., 2018). In patients with Len-refractory MM, the combination of pomalidomide, cyclophosphamide, and Dex confer a superior ORR and PFS than pomalidomide and Dex (NCT01432600) (Baz et al., 2016). In ALL, treatment with retinoid receptor agonists is able to fortify the sensitivity of IKZF1-aberrant BCR-ABL1 ALL patients to tyrosine kinase inhibitor therapy (Churchman et al., 2015).

## 5 Conclusion

Ikaros and its analogues play an important role in regulating normal lymphopoiesis, immune diseases, and generation of some tumors. In terms of categories, the Ikaros protein family consists of five members named Ikaros, Helios, Aiolos, Eos, and Pegasus. Ikaros acts both as a transcriptional repressor and as an activator by binding to assorted nuclear factors referred to epigenetic regulation and chromatin remodeling in different malignancies. And its own activities are thought to be regulated by post-translational phosphorylation, SUMOylation, and ubiquitination. Studies on signaling pathways about Ikaros have clarified that the preBCR signal pathway, Notch pathway, integrin signaling pathway, and IRFs are related to Ikaros signal transduction. For lymphocytes in healthy subjects, the Ikaros protein family exerts different effects on the growth, reproduction, and differentiation of many kinds of innate or adaptive lymphocytes *in vivo* and prevents autoimmunity by administering BCR unresponsiveness and repressing TLR signaling transduction.

Ikaros appears to function as a transcriptional inhibitor and activator by binding to different nuclear factors related to epigenetic regulation or targeting genes directly. The Ikaros-HDAC1 complex, inducing the formation of inhibitory chromatin, suppresses the transcription of its target genes accordingly. Ikaros has an adverse effect on the development of leukemia *via* globally regulating the enhancer or super-enhancer landscape and pioneer ring activity. Analogically, Ikaros is able to directly act on some target genes, such as those that activate the target genes’ transcription, cause transcription suppression, determine cell cycle progression, control cell survival, and develop drug resistance. Ikaros induces the powerful upregulation of G6PD, effecting malignancies through glucose metabolism.

IMiDs lead to selective ubiquitination and proteasomal degradation of Ikaros and Aiolos through recruiting TFs to the CRL4^CRBN^ E3 ubiquitin ligase, achieving the purpose of therapy for MM and other malignancies. Novel thalidomide analogs, are currently in clinical trials as a monotherapy and in combination with other drugs, illustrating that CELMoDs have strong antitumor and immunostimulatory capabilities in patients with hematological malignancies. Even so, developing resistance to administered therapies is a huge obsession. Combination therapy and developing drugs against new targets may be potential treatments. Other treatments, such as protease kinase inhibitors, monoclonal antibodies, and phytochemicals were also applied to patients with IKZF-missing phenotypes, but the efficacy remains to be verified.

## Glossary

TF: transcription factor
IKZF: IKAROS family zinc finger
AML: acute myeloid leukemia
Len: lenalidomide
MM: multiple myeloma
BCP-ALL: pediatric B-cell precursor acute lymphoblastic leukemia
ALL: acute lymphoblastic leukemia
CRBN: cereblon
MCL: mantle cell lymphoma
IMiDs: immunomodulatory drugs
MDS: myelodysplastic syndrome
CLL: chronic lymphocytic leukemia
NuRD: nucleosome remodeling and deacetylase complex
HDAC: histone deacetylase complexes
PP1: protein phosphatase 1 protein phosphatase 1α
PI3K: phosphatidylinositol-3 kinase
BCR: B-cell receptor
TCR: T-cell receptor
IRF: interferon regulatory factor
SUMOylation: small ubiquitin-related modifier
RUNX: runt-related transcription factor family
CK2: carcinogenic casein kinase II
ILC: innate lymphoid cell
TH: T helper cell
Treg: T regulated cell
KDM5B: lysine [K]-specific demethylase 5B
PHF2: PHD finger protein 2
ARID5B: AT-rich interactive domain-containing protein 5B
G6PD: glucose-6-phosphate dehydrogenase
JAK-STAT: Janus kinase-signal transducer and activator of transcription
IL7R: interleukin-7 receptor
BCL6: B-cell lymphoma 6
BACH2: basic leucine zipper transcription factor 2
RAG: recombination-activating gene
KLF2: Krüppel-like factor 2
Blimp-1: B lymphocyte maturation inducing protein-1
CAR T-cell: chimeric antigen receptor T-cell
CELMoDs: CRBN E3 ligase modulating drugs
SLL: small lymphocytic lymphoma
RRMM: relapsed/refractory multiple myeloma
NDMM: newly diagnosed multiple myeloma
BTZ: bortezomib
BTK: Bruton tyrosine kinase
BRD4: bromodomain-containing protein 4
PAX5: paired box 5
